# Comparative Transcriptomic Analysis of Vernalization- and Cytokinin-Induced Floral Transition in *Dendrobium nobile*

**DOI:** 10.1038/srep45748

**Published:** 2017-03-31

**Authors:** Zhenzhen Wen, Wenzhong Guo, Jinchi Li, Haisheng Lin, Chunmei He, Yunquan Liu, Qunyu Zhang, Wei Liu

**Affiliations:** 1 College of life sciences, South China Agricultural University, Guangzhou, 510642, China; 2 Guangdong Engineering Polytechnic, Guangzhou, 510520, China; 3 State Key Laboratory for Conservation and Utilization of Subtropical Agro-bioresources,South China Agricultural University, Guangzhou, 510642, China

## Abstract

Vernalization is required for floral initiation in *Dendrobium*. Interestingly, those beneficial effects can also be achieved by exogenous cytokinin application in greenhouses. Thus, an as yet unknown crosstalk/interaction may exist between vernalization and cytokinin signaling pathways. In this study, we showed, by *de novo* transcriptome assembly using RNA-seq data from both vegetative and reproductive tissue samples, that some floral transition-related genes—*DnVRN1, FT, SOC1, LFY* and *AP1*—were differentially expressed in low-temperature-challenged (LT) or thidiazuron (TDZ)-treated plants, compared to those mock-treated (CK). Both LT and TDZ upregulated *SOC1, LFY* and *AP1*, while the upregulation of *DnVRN1* and *FT* was only LT-induced. We further found that LT promoted the upregulation of some key cytokinin signaling regulators, including several cytokinin biosynthesis-related genes and type-B response regulator (RR)-encoding genes, and that both LT and TDZ triggered the significant upregulation of some marker genes in the gibberellin (GA) signaling pathway, indicating an important low temperature-cytokinin-GA axis in flowering. Our data thus have revealed a cytokinin-GA signal network underlying vernalization, providing a novel insight into further investigation of the molecular mechanism of floral initiation in *Dendrobium*.

Vernalization is the process of promoting flowering in certain plants by prolonged exposure to the cold of winters. In *Arabidopsis*, vernalization represses the expression of *FLOWERING LOCUS C (FLC*), a potent flowering repressor, and this suppression requires VERNALIZATION INSENSITIVE 3 (VIN3)^1^, VERNALIZATION 2 (VRN2)^2^, and VERNALIZATION 1 (VRN1)^3^. As a MADS-box transcriptional regulator, FLC inhibits floral initiation by reducing the expression of flowering-time integrators including *FLOWERING LOCUS T (FT*) and *SUPPRESSOR OF OVEREXPRESSION OF CONSTANS 1(SOC1*). Vernalization-induced FT and SOC1 then activate floral meristem-identity genes such as *LEAFY (LFY*) and *APETALA 1 (AP1*), eventually leading to floral initiation.

Cytokinins, an important class of phytohormones, have long been considered to play a role in floral transition^4^. Two recent reports have demonstrated that endogenous cytokinin level is significantly increased in vernalization-induced reproductive development of *Brassica napus* plants^5^, and that exogenous application of N^6^-benzylaminopurine (BAP), a synthetic cytokinin, promotes flowering in *Arabidopsis* grown in non-inductive short days through transcriptional activation of the *FT* paralogue *TSF* and *SOC1*^6^. In addition, by transcriptomic analysis cytokinins have been found to interplay with gibberellins (GA) in producing systemic flowering signal(s) in leaves of *Glycine max*^7^.

*Dendrobium* is one of the largest genera of the *Orchidaceae* and consists of more than 1200 species^8^, most of which are important commercial orchids that account for 20% of total orchid sales. They fall into two categories, *nobile*-type and *phalaenopsis-*type, which in horticulture are usually sold as potted and cut flowers, respectively^9^. The potted *nobile*-type *Dendrobium* requires moderate low temperatures (vernalization) for flower induction, except for some cultivars that are regulated by photoperiod^10^,^11^. While cold treatment is effective, its effects are of regional variations. To meet market demand, a growing interest has been shown in the control of flowering time. Cytokinin treatment was demonstrated to stimulate flowering as early as 1978 in a sympodial orchid hybrid *Dendrobium* Louisae^12^, but the underlying molecular mechanisms remain largely unknown.

We recently developed an effective method of flowering time control in *nobile*-type *Dendrobium* using thidiazuron (TDZ), a potent synthetic cytokinin-like compound, by which plants flowered 10~15 days earlier than cold-treated plants, even in the absence of cold in greenhouses^13^. Based on this fact, we deduce that an unknown crosstalk/interaction may exist between vernalization and cytokinin signaling pathways sustaining the floral initiation in *D. nobile*. To explore this underlying molecular mechanism, in this study differential gene expression profiles were investigated in the transcriptomic scope of the wild-type, cold- and TDZ-treated plants. This transcriptomic analysis was based on the *de novo* transcriptome assembly platform, Trinity^14^, using RNA-seq data from both vegetative (pseudobulb, leaf and root) and reproductive (axillary bud) tissue samples. Our data suggest an important low temperature-cytokinin-GA axis regulating flowering time of *D. nobile*.

## Results

### De novo assemblies of the transcriptomes of D. nobile plants

Two-year old adult plants were randomly selected and exposed to 4 °C (LT-group), given one-time spray of 20 mg L^−1^ of TDZ (TDZ-group), or left untreated (control group) for 30 days. Leaves, roots, pseudobulbs, and axillary buds of each group were collected and pooled at the beginning and every five days of this duration. Total RNA was then extracted for RNA-seq using an IlluminaHiSeq™ 2000 platform. The subsequent *de novo* transcriptome assemblies using the Trinity platform^14^, which was based on the 110 M raw reads obtained from the RNA-seq, thus yielded 99,686 unigenes, including 38,309 clusters and 61,377 singletons (Table 1). The average unigene size was 684 bp, and 21% of the unigenes were greater than 1000 bp in length (Figure S1).

### Functional annotation and classification of D. nobile Unigenes

Unigene annotation were performed by BlastX against the protein databases NR, Swiss-Prot, KEGG and COG (E-value < 1e-5). The annotated unigenes accounted for 53.9% of all the assembled unigenes. Of these 53,685 annotated unigenes, 20,751 fell into 25 COG functional categories (Figure S2) including the “signal transduction mechanisms” (13.4%), and 37,784 were annotated to 53 GO terms (Figure S3) including the “reproduction” (11.3%) and “signaling” (8.2%). By that GO enrichment analysis, we also noticed that some unigenes were associated with nucleic acid binding transcription factor activity (2%), representing previously reported chromatin modifications in floral transition^15^. To further identify the biological pathways in which the annotated unigenes get involved, 26,624 annotated unigenes were mapped to 125 reference canonical pathways in the KEGG pathway database (Table S2), among which 1,305 unigenes (4.33%) were assigned to “plant hormone signal transduction” and 336 unigenes (1.11%) were grouped as “zeatin biosynthesis”.

### Homologs in relation to floral transition

Based on the above functional prediction and enrichment analysis, we identified some *D. nobile* homologs of *Arabidopsis FT, SOC1, LFY* and *terminal flower 1 (TFL1)*^16^, which are the key flowering-time integrator genes (Table S3). *FLC* in *Arabidopsis*, and *VRN1, VRN2*, and *VRN3* (cereal *FT*) in temperate cereals^17^,^18^ are well known as critical vernalization-induced flowering regulators. However, as shown in a recent report^19^, *FLC* and *VRN2* homologs were not found in our dataset, but the *D. nobile VRN1 (DsVRN1*) (unigene ID: CL16305.Contig1_TRA) identified, supporting the difference demonstrated in flowering control between dicot and monocot plants^18^. Many genes related to chromatin regulation were also identified, which showed that, like the vernalization pathway in *Arabidopsis*, cold and cytokinin may use chromatin regulators to control flowering time in *D. nobile*. There were 32 unigenes annotated as genes encoding zinc finger protein *CONSTANS* (Table S4), the first B-box protein identified in *Arabidopsis* playing a central role in the photoperiodic flowering pathway^20^.

**Figure 1:**
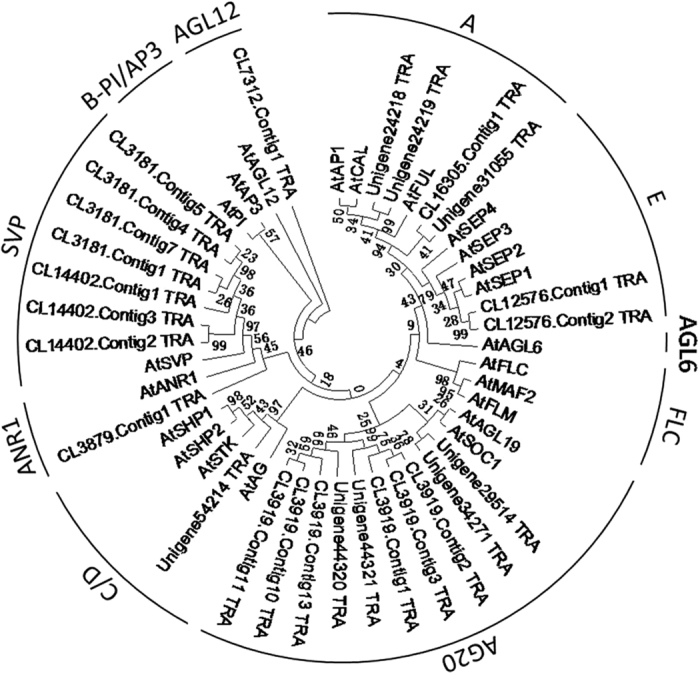
Molecular phylogenetic analysisof MADS-box genes identified from *D. nobile* transcriptome. Evolutionary analyses based on conserved MADS-box domains were conducted in MEGA5 using the maximum likelihood method. The bootstrap consensus tree inferred from 300 replicates is taken to represent the evolutionary history of the taxa analyzed. The analysis involved 26 MADS-box domain containing genes identified in this study and 22 MADS-box genes from *Arabidopsis* under gene bank accession number (GI) as shown in Table S9.

Additionally, 64 unigenes encoding MADS-box transcription factors were identified, including the homologs of important flowering time genes such as *SVP, SOC1* and *AP1*/*VRN1* (cereals) (Table S5). Of these unigenes, 26 had a conserved MADS-box domain, and 24 of them had a K-box domain following the MADS-box domain. The phylogenic tree constructed based on the MADS-box domain is shown in Fig. 1. The 26 putative MADS-box transcription factors were mainly classified into *AP1/FUL, AGL20*-like, E-, C/D-, and *SVP* clades, while *FLC*-like genes were absent in the current transcriptomic analysis.

### Homologs in relation to cytokinin metabolism and signaling

The enzymes encoded by *IPT, CYP* and *LOG* catalyze the rate-limiting step in cytokinin biosynthesis, and *CKX* encodes the key enzyme for cytokinin metabolism^21^. Homologs of *IPT, CYP* and *LOG* were all identified in the *D. nobile* transcriptome (Table 2), and there were seven unigenes annotated as *CKX.* Response regulators (RRs) are the downstream nuclear-localized components of the two-component system that are believed to be the major regulators of cytokinin response in plants^22^. There are 22 RRs in *Arabidopsis*, which were originally divided into two large classes (type-A and type-B) based on protein sequences, domain structures and transcriptional induction by cytokinin. Type-B RRs appear to act as the positive regulators of cytokinin signaling, while type-A RRs act as the negative regulators, forming a negative feedback loop. The homologs of both type-A and type-B RRs were identified in our dataset, and four of them were validated by real-time PCR (Table 2). All of these cytokinin-associated genes would helpful for us to investigate the mechanism of cytokinin induced floral initiation in *D. nobile*.

### Differentially expressed genes in cold-treated plants

Total RNAs were extracted from leaves, pseudobulbs, axillary buds and roots collected and then mixed in a mass ratio of 1:1:1:1. Mixed RNAs from the control, cold-treated, and TDZ-treated *D. nobile* plants were used to construct three DGE libraries and then sequenced. The sequencing quality evaluation and alignment statistics are shown in Table S6. The percentage of all low quality reads containing only adaptors or N reads was less than 2%. After filtering the low quality tags, the total numbers of clean tags in the control, cold-treated, and TDZ-treated libraries were 12.23, 11.73 and 11.93 million, respectively. The number of tags that could be mapped to the transcriptomic sequences amounted to 10.17 (83.15%), 9.79 (83.52%), and 10.11 (84.79%) million respectively. The number of clean ta for each gene was calculated, and the genes that were differentially expressed were identified according to the methods described by Audic and Claverie^23^.

A total of 2,361 differentially expressed genes, including 1,060 up-regulated and 1,301 down-regulated genes, were identified in the cold-treated materials. The expression of nine genes, including a membrane-associated type I inositol 1,4,5-trisphosphate 5-phosphatase encoding gene (CL10301.Contig2_TRA) and a cell-wall structure protein encoding gene (CL3912.Contig1_TRA), was completely inhibited by cold, while a total of 40 genes were activated (Table S7). Based on GO functional classification (Figure S4), most of the gene sets regulated by cold were mainly correlated to cellular and organelle component organization.

**Figure 2:**
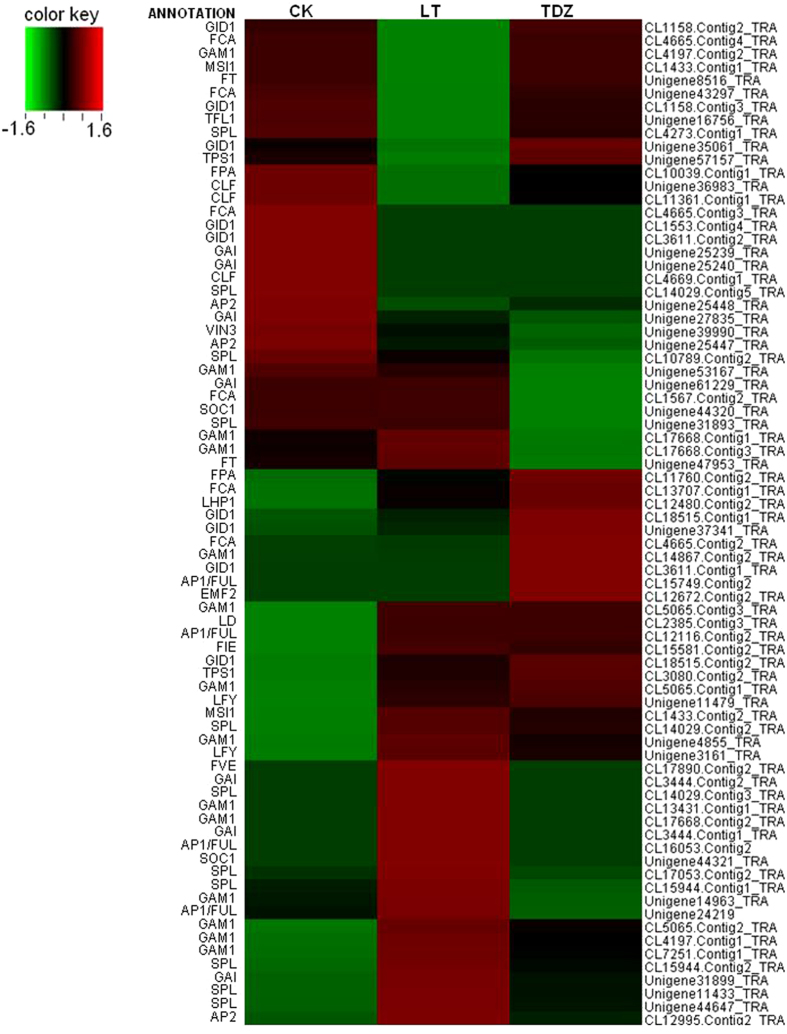
The regulatory pattern of the 76 candidate genes for flowering time in *D. nobile.* Each column represents an experimental sample (CK-control, LT-cold treatment, and TDZ-TDZ treatment), and each row represents a gene, the annotation of which were listed on the left. Expression differences were determined by RPKM (Reads Per kb per Million reads) values calculated according to the method provided by Mortazavi and Williams (2008), and shown in different colors. Red indicates high expression, and green indicates low expression.

**Figure 3:**
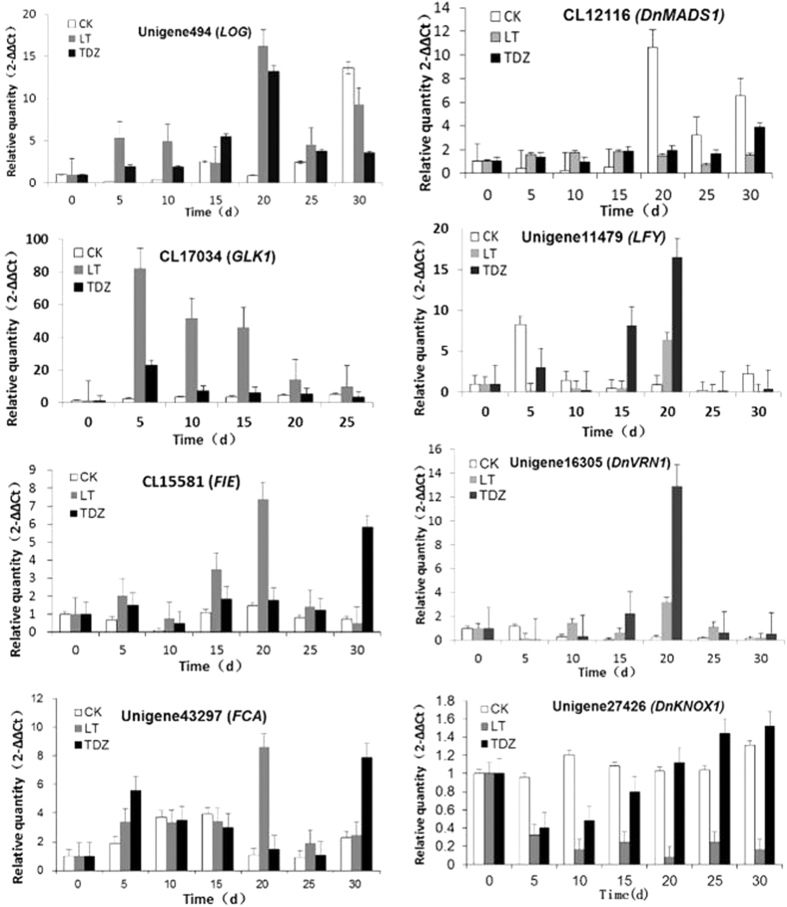
qRT-PCR analysis of expression variation of some candidate flowering-time genes. The relative quantity of gene expression in axillary buds of control, cold-treated and TDZ-treated plants were calculated compared with the control gene *DnActin*. And represented by CK, LT, and TDZ, respectively, which showed in the figure marked by different color. Error bars indicate the range of variation in three biological repeats.

Cluster analysis of flowering-time associated genes was conducted. Two homologs of *FT*, Unigene 8516 and Unigene 47953, were regulated by cold. Unigene 8516, annotated as *DnTSF*^6^ by sequence blast, was inhibited by cold, whilst unigene 47953, a gene sharing high homology with *DnFT* identified by Liang *et al*.^19^, was obviously up-regulated by cold. Transcription of the homolog of *SOC1*, another floral integrator, was apparently activated by cold, too (Unigene44321, Fig. 2). The results showed that, the expression of floral identity genes *LFY* and *AP1* was up-regulated, while that of upstream repressor *MSI1* and *CLF*^16^ was down-regulated (Fig. 2). Real-time PCR using axillary buds showed the expression of *AP1* and *LFY* peaked at 20 d post-vernalization (Fig. 3), and hit the bottom at 5 d after the completion time of floral transition reported by Liang *et al*.^19^. As noted previously, there were no homologs of *FLC* identified in this study. The unigene CL16053.contig2_TRA, a homolog of *Arabidopsis AP1*, was identified as *DnVRN1*, the cereal *VRN1* homolog in *D. nobile*. Its expression was significantly up-regulated by cold. Another 12 transcripts with homology to *AP1* were also regulated by cold, and the expression of the homologs of *AG, AGL16, AGL21, SVP* and *ZMM17* were regulated by cold as well (Table 3). All of these MADS-box genes were candidates for further functional analysis to clarify the underlying mechanism of cold-induced floral initiation. Besides, *CLF, ULT1* and *REF6* are important members in chromatin methylation of *FLC* and *VRN1*, their homologs were also significantly regulated by cold in *D. nobile*.

**Figure 4:**
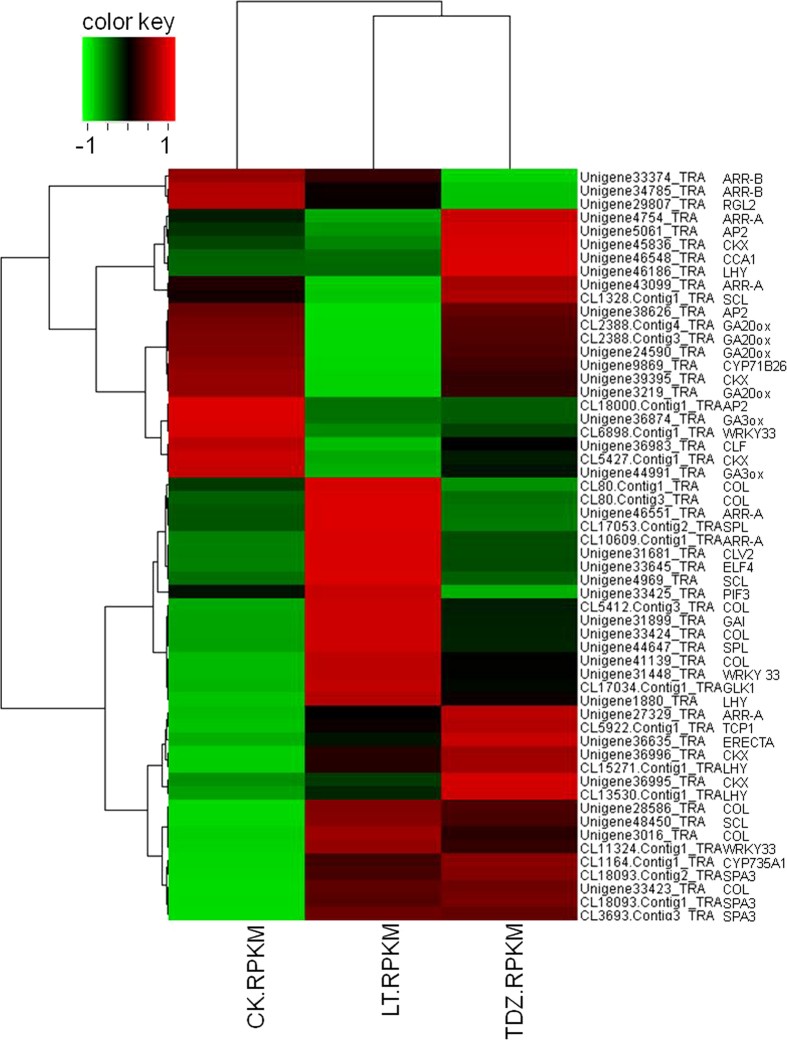
Hierarchical cluster analysis of differentially expressed genes (DEGs) in cold- and TDZ-treated plants. Each column represents an experimental sample (CK-control, LT-cold treatment, and TDZ-TDZ treatment), and each row represents a gene. 54 DEGs that were significantly regulated (p-value < 0.05) were analyzed in pairwise comparisons (CK-VS-LT, CK-VS-TDZ,and LT-VS-TDZ). Expression levels were determined by RPKM (Reads Per kb per Million reads) values calculated from Illumina read counts, according to the method provided by Mortazavi and Williams (2008). Expression differences are shown in different colors. Red indicates high expression, and green indicates low expression.

Results above showed a somewhat conserved molecular mechanism of vernalization in *D. nobile*. Considering the objective of this study, we analyzed the expression profiles of the cytokinin-associated genes. The homologues of *IPT* and *CYP735A1*, which encode the two key enzymes for cytokinin biosynthesis, were activated by cold (Table S8). Expression levels of type-B RRs in cold-treated plants increased, while those of type-A RRs were down-regulated (Table S8, Fig. 4), showing an improved cytokinin signal transduction.

Interes, we found that the transcription of six genes which shared homology with the cereal transcription factor GAMyb (*GAM1*) was significantly up-regulated by cold (Fig. 2). *GAM1* is an early GA response gene encoding a key molecule in synthesizing and secreting hydrolytic enzymes in cereal aleurone^24^. The results indicated the nutrition metabolism regulated by a cold-induced GA signal. But what was contradictory to this was that the down-regulated expression of the GA receptor GID1 encoding genes, and the activation of DELLA protein GAI encoding genes in the cold-treated plants (Fig. 2).

**Figure 5:**
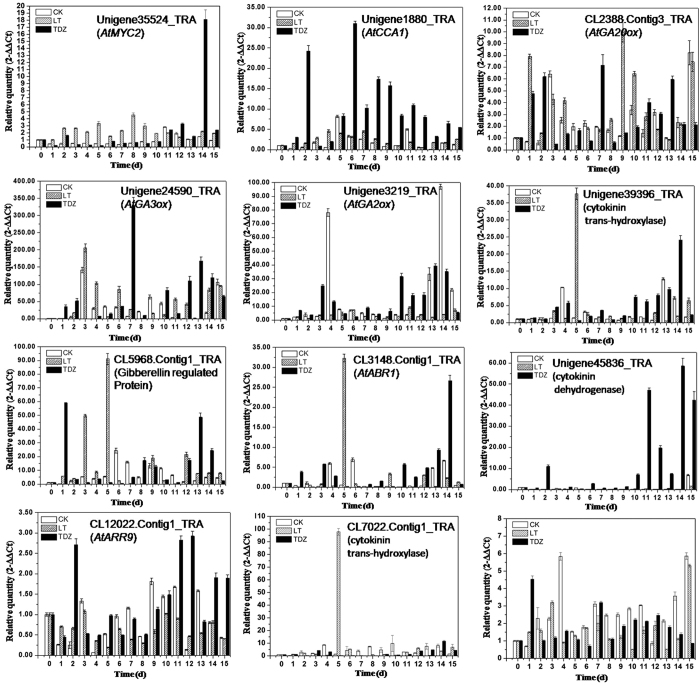
qRT-PCR analysis of expression variation of GA-associated genes over time during the course of cold (LT) and TDZ treatments. The relative quantity of gene expression in axillary buds of control (CK), cold-treated (LT) and TDZ-treated (TDZ) plants were calculated compared with the control gene *DnActin*. Error bars indicate the range of variation in three biological repeats.

The roles of cytokinin and GA in cold-induced flowering were further revealed by real-time PCR, using RNAs extracted from axillary buds. The *GA20ox* homolog (CL2388.Contig3) was immediately up-regulated by cold, and the transcription of the *GA3ox* homolog (Unigene24590_TRA) obviously increased at 4 d. The results indicated a cold-regulated GA signaling, along with the fact that the transcription of the GA-regulated protein encoding cDNA (CL5968.Contig1_TRA) increased at 3d and 4d post-vernalization. The transcription of *GA20ox* homologue decreased from 2d after treatment and hit the bottom at 5d, and the *GA3ox* homologue was inhibited at the same time (Fig. 5). In addition, the relative transcriptional level of Unigene494_TRA obviously increased at 5d, 30d and peaked at 20d after cold-treatment (Fig. 3). This gene was annotated as the homolog of *AtLOG8*, which encodes the key enzyme of the last step of cytokinin activation in *Arabidopsis*. In accordance with this, an expression peak of two cytokinin trans-hydroxylase encoding genes, Unigene39396_TRA and CL7022.Contig1_TRA, was observed at 5 d (Fig. 5), which showed an up-regulation of cytokinin biosynthesis induced by cold during this time. Besides, another obvious activation of *GA20ox* and *GA3ox* homologues appeared at 9 d and 11 d, respectively. These changes together with the inhibition of *GA2ox* homologue (Unigene3219_TRA) at 14d indicated an activation of GA signaling during the late stage of the cold-induced floral transition.

### Differentially expressed genes in TDZ-treated plants

The analysis of DGE sequencing resulted in 588 up-regulated genes and 693 down-regulated genes in the TDZ group compared to the control group, which was half as many as the genes regulated by cold. The nine genes completely inhibited by cold were also inhibited by TDZ. Five genes including a starch phosphorylase encoding gene (Unigene13605_TRA) was activated by TDZ (Table S7), and the transcription of the two *TPS1 (Trehalose-6-P synthase1*) homologues (Unigene57157_TRA, CL3080.Contig2_TRA) were up-regulated (Fig. 2). It was reported that *TPS1* was regulated by sucrose, and activated the expression of *FT* in leaves through the miR156 pathway^25^. The activation of a starch phosphorylase encoding gene and *TPS1* homologues indicated the nutrition metabolism involved in TDZ-induced flowering.

**Figure 6:**
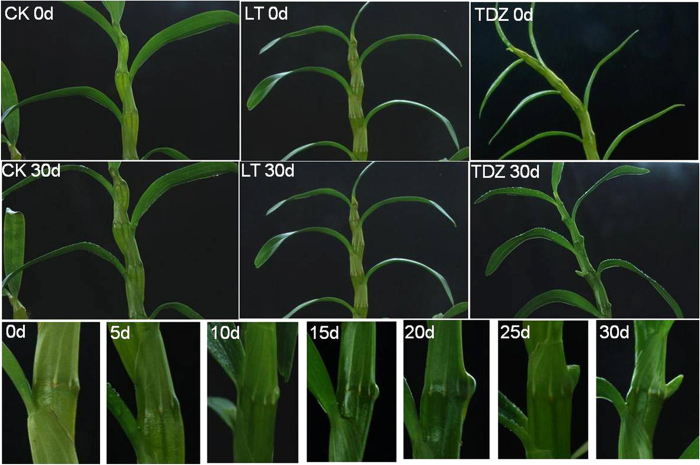
Morphology changes of axillary buds of *D. nobile* over time during the course of cold (LT) and TDZ treatments. Pictures of control plants (CK), cold-treated (LT) and TDZ-treated plants before treatment (0 d) and at 30 d after treatment were showed, no apparent morphology changes of axillary buds observed in control and cold-treated plants. But an obvious development of inflorescence appeared in TDZ-treated plants. And the close-up observance of axillary buds at 0, 5, 10, 15, 20, 25, and 30 d after TDZ-treatment showed that the buds bulged at 15 d, the bottom panel.

Homologues of the floral integrator *SOC1* were up-regulated in TDZ-treated plants, but apparent changes of CL16053.contig2_TRA (*DnVRN1*) were not observed (Table 3). The floral meristem identity genes *LFY* and *AP1* were activated (Fig. 2, Table 3), indicating the complete floral transition by TDZ. Results of the real-time PCR analysis showed that the transcription of *AP1* and *LFY* peaked at 20 d after TDZ treatment, as in the cold-treated axillary buds, and their transcriptional level were much higher than that in the cold-treated plants. Consistent with these real-time PCR results, the axillary buds of the TDZ-treated plants were observed to swell and bulge out about 1–2 cm (Fig. 6). Unlike the subsequent flower development of the cold treated plants occurring under warmer conditions, the TDZ-induced floral initiation was followed by inflorescence and floral organ development, consistent with the observation of the increased transcription of ERECTA encoding cDNAs (Fig. 4). The latter play important roles in meristem differentiation and floral organ development.

There was no surprise to observe the feedback inhibition of the endogenous cytokinin level in the TDZ treated *D. nobile*, which was supported by the up-regulation of cytokinin dehydrogenase (CKX) encoding genes. CKX is a key enzyme functioning in cytokinin catabolism. Besides, type-A RRs which negatively regulate cytokinin signaling were significantly up-regulated by TDZ, while the positive regulators type-B RRs were down-regulated (Fig. 4, Table S8). The expression of a *CKX* homologue, Unigene45836_TRA, increased significantly at 2 d post-application of TDZ, and maintained at a low level in a few days, then increased at 11 d again. These changes were consistent with the upregulation of CL12022.Contig1_TRA, a type-A RR encoding cDNA (Fig. 5), indicating the decreased endogenous cytokinin biosynthesis.

We also noticed that the transcription of GA receptor gene *GDI1* was up-regulated by TDZ, and the DELLA protein GAI and RGL encoding genes were down-regulated (Figs 3 and 4), suggesting the enhanced GA signaling induced by TDZ. Indeed, the GA-regulated protein encoding cDNA, CL5968.Contig1_TRA, was significantly up-regulated by TDZ at 1d, along with the transcriptional activation of the *GA20ox* homologue at 7d and the CL5986.Contig1_TRA at 8d (Fig. 5).

## Discussion

*D. nobile* is one of the most widespread ornamental members of orchid family, but the control of flowering time still remains a bottleneck in production. Our data in this study has demonstrated that the exogenous application of TDZ may effectively induce flowering in *D. nobile* grown in greenhouses, thus providing a cost-effective method for floral transition in horticulture. Cytokinins were proved to be effective in inducing flowering in some plants, but there are few reports on the crosstalk between vernalization and cytokinin signaling pathways. The molecular mechanism sustaining the TDZ-induced floral transition needs to be clarified.

There are very limited genetic information of *D. nobile*, except for the recently reported expressed sequence tags (ESTs) from flowering buds of *Orchidaceae* species^26^ and the genomic sequences of *Phalaenopsis equestris*^27^ and *Dendrobium officinale*^28^. The cDNA data acquired in this study was much more than that acquired from the transcriptomes of other orchids, *i.e. oncidium*^29^, *cymbidium*^30^, and *Phalaenopsis*^31^. All the flower development-related genes identified were valuable for further investigation of the floral transition in *D. nobile.* Starting at this point, we studied the molecular mechanism underlying the crosstalk between vernalization and TDZ-induced floral initiation by comparative transcriptomic analysis.

The up-regulation of *DnVRN1*, floral integrator *FT* and *SOC1*, and flower identity genes *AP1* and *LFY* by cold, together with the differential expression of *CLF, REF6* and many chromatin regulation associated genes, showed a somewhat conserved molecular mechanism of flowering used by *D. nobile*. Unexpectedly, except for the up-regulation of *AP1* and *LFY*, we find little flowering-time associated genes similarly regulated by cold and TDZ. But the membrane-associated type I inositol 1,4,5-trisphosphate 5-phosphatase encoding gene (CL10301.Contig2_TRA), and the cell-wall structure protein encoding gene (CL3912.Contig1_TRA) were inhibited by both cold and TDZ. These two genes are both regulators of cell wall synthesis. These results indicated that cell wall reorganization was involved in floral initiation induced by cold or TDZ in *D. nobile*, as reported in Arabidopsis^32^. An extensive overlap between cytokinin and glucose-signaling pathways was proposed by Kushwah and Laxmi^33^, and the regulation of *TPS1* by TDZ in this study suggested a role of sugar signaling in flowering of *D. nobile*. Our data also support the roles of sugar signaling in cold-induced floral initiation by exhibiting the cold-induced upregulation of a GA-response gene *GAM1*, which encodes a key molecule involved in synthesizing and secreting hydrolytic enzymes in cereal aleurone^24^.

By the comparative transcriptomic analysis shown in this study, we demonstrated that GA signaling was significantly improved during both vernalization and TDZ induced floral transitions. GA was proved to play important roles in floral initiation but not in flower development, and many reports have pointed out that vernalization makes plants more sensitive to GA^34^. Recently, it has been revealed that DELLA proteins, the repressors of GA signaling, interact with FLC in vernalization-induced flowering^35^. The positive effects of GA on the termination of vegetative development have been reported in *Arabidopsis*^36^, but still remain unexplored in *D. nobile*. According to the transcriptomic analysis presented in this study, a crosstalk between GA and cytokinin contributes to vernalization-induced floral initiation. The temporal transcriptional activation of the GA-response genes appeared immediately after the cold treatment, and in a latter stage, 7–9 d post-vernalization. In these two stages, cytokinin signaling was also enhanced, which was supporting by the significant up-regulation of cytokinin synthase encoding gene (Unigene39396_TRA, CL7022.Contig1_TRA, Fig. 3), and the transcriptional changes of type-A RR and type-B RR genes. Consistent with these data from the cold-treated plants, an enhanced GA signaling was also observed in the TDZ group with the fact that the TDZ application induced a ten-fold increase in the expression levels of *CKX, GA3ox* and *GA20ox* at 2 d, the peak expression of *GA3ox* and *GA20ox* at 7d (Fig. 5), together with the up-regulation of *GID1* and down-regulation of DELLA protein GAI and RGL encoding genes (Figs 2 and 4). Interestingly, the expression of Unigene45836_TRA (*CKX*) was also significantly increased in a latter stage, indicating the sustaining cytokinin signaling activation in the late stage of the duration of TDZ treatment (Fig. 5).

Morphology analysis of the axillary buds showed that swelling appeared at 10d after TDZ application, while no changes were observed in the LT-treated plants (Fig. 6). In *Glycine max*, by transcriptomic analysis it was indicated that cytokinins could promote flowering by cytokinin signaling itself through B-type RRs, or play negative roles via inhibiting GA, which may activate the flowering integrator *SOC1*^37^. AP1 may suppress the biosynthesis and activate the degradation of cytokinin in the establishment of determinate floral meristems in *Arabidopsis*^38^, which indicates that cytokinins function early in floral transition. According to the results of this study, it can be deduced that the rapid accumulation of cytokinins in meristems can be induced by the application of TDZ, facilitating the positive effects of cytokinins imposed on floral initiation. Meanwhile, the feedback inhibition of endogenous cytokinin level may result in the enhancement of GA signaling, which was important for further flower development. For the vernalized plants, the enhanced GA signaling induced by cold was followed by the activation of cytokinin biosynthesis genes at 5d (Fig. 5), leading to the floral initiation similar to that in the TDZ treated plants. The inhibitory effects of GA on cytokinin during vernalization may cause a delay of flowering compared to the TDZ-treated plants. More details and the molecular mechanism of the subsequent flower development need to be further investigated.

## Methods

### Plant materials, growth conditions, cold- and TDZ-treatment

Plants of *D. nobile* were grown without regulation of photoperiod in a greenhouse at the College of Life Sciences at South China Agricultural University (China). Two-year old adult plants were selected on Oct. 9, 2012, and 200 of them were moved to an air-conditioned storage room with a temperature setting of 4°Cduring the night (7:00 p.m. to 7:00 a.m.) for 30 d. Another 400 plants were divided into two groups, one of which was sprayed with 20 mg L^−1^ of TDZ. All of the plants were watered every week. Organs (i.e., leaf, pseudobulb, root, and axillary buds) from cold-treated, TDZ-treated, and control plants were sampled at 0 d, 5 d, 10 d, 15 d, 20 d, 25 d, and 30 d after treatment. The number of plants sampled was 20 every time for every treatment. All samples were frozen in liquid nitrogen and stored at −80 °C.

### RNA extraction, library preparation, and Illumina sequencing

Total RNA was extracted from sampled tissues using a Qiagen RNeasy Kit according to the manufacturer’s instructions. Total RNA extracted from four organs (i.e., leaf, pseudobulb, root, and axillary buds) at different time from one treatment were mixed at an equal mass ratio, and then acquired 3 RNA samples from cold-treated, TDZ-treated and control plants, respectively. For transcriptome sequencing, 3 samples were mixed at a mass ratio of 1:1:1, then mixed total RNA was used for messenger RNA isolation with polyA selection and subsequent library construction using the TruSeq RNA sample preparation protocol from Illumina (San Diego, CA). Two biological replicates were sequenced and analysed for each of the nine tissue-treatment combinations. Single-end sequencing was performed on the nine samples by the Illumina HiSeq™ 2000 platform, and raw sequence data generated is available for download at the NCBI Sequence Read Archive under number SRR4169965.

### Functional annotation of the D. nobile transcriptome

After filtering of raw reads, *de novo* assembly of the transcriptome was carried out with Trinity^14^, a short reads assembly program. The generated unigenes were used for functional annotation against databases, including non-redundant (nr), SwissPort, COG, KEGG, and GO with a cut-off E-value of 0.00001.

### Differential gene expression (DGE) analysis with D. nobile transcriptome

Total RNA extracted from four organs (i.e., leaf, pseudobulb, root, and axillary buds) at different time from one treatment were mixed at an equal mass ratio, and then acquired 3 RNA samples from cold-treated, TDZ-treated and control plants, respectively. The samples were used for RNA sequencing analysis via Illumina HiSeq™ 2000. After removal of low quality tags, empty tagsand tags with only one copy number, the raw DGE data (CK, LT, and TDZ),which were deposited in the GeneBank Short Read Archive (accession number SRR4266569), were mapped to the *do novo* assembled transcriptome of *D. nobile*. The gene expression level is calculated by using RPKM^39^ method, and the genes that were differentially expressed between the two samples were identified according to methods described by Audic and Claverie^23^.The false discovery rate (FDR) was used to determine the threshold P-value in multiple tests. We used FDR < 0.001 and an absolute value of the log2 ratio > 1 as the threshold to determine significant differences in gene expression. Differentially expressed genes were used for GO and KEGG enrichment analyses with a corrected P-value ≤ 0.05 as a threshold. Cluster analysis of gene expression patterns was performed with Cluster^40^ and Java Treeview^41^ software.

### Gene expression variety analyzed by quantitative real-time PCR

A 500 ng sample of total RNA from axillary budswas used for reverse transcription using Quanta qScript cDNA SuperMix, and the cDNA was diluted 20 times before it was used for analysis by quantitative real-time PCR (qPCR). Each reaction consisted of 2 μl 20-fold cDNA, 9 μl SYBR-Green supermix, 1 μl of 6 μM forward and reverse primers, and 7 μl of sterile water. qPCR was performed in an Eppendorf Mastercycler ep realplex with the following parameters: one cycle of 2 min at 95 °C, followed by 40 cycles of15 s at 95 °C, 45 s at 57 °C, and 35 s at 68 °C. The last step for each reaction was a melting curve generation to test amplicon specificity. All qPCR reactions were performed in three technical and three biological replicates. All samples were compared with the control gene *DnActin*. The primer sequences for genes that were verified through qPCR are presented in Supplementary Table S1.

## Additional Information

**How to cite this article:** Wen, Z. *et al*. Comparative Transcriptomic Analysis of Vernalization- and Cytokinin-Induced Floral Transition in *Dendrobium nobile.* *Sci. Rep.* **7**, 45748; doi: 10.1038/srep45748 (2017).

**Publisher's note:** Springer Nature remains neutral with regard to jurisdictional claims in published maps and institutional affiliations.

## Supplementary Material

Supplementary Information

## Figures and Tables

**Table 1:** Summary statistics of orchid transcriptome.

Species	*D. nobile*	*O.* Gower Ramsey	*C. sinense*	*Phalaenopsis*
Sequencing platform	Illumina	454 GS-FLX	Illumina	454 GS-FLX
Total number of raw reads	110,000,000	920,196	59,512,598	42,034,787
Total number of clean reads	95,051,256	916,682	54,248,006	206,960
Total clean nucleotides (nt)	8,554,613,040	301,843,196	4,882,320,540	47,186,880
Average read length	90	327	90	228
Total number of contigs	255,369	120,219/50,908	147,162	34,630/8233
Mean length of contigs (nt)	269	267/493	326	201/364
Total number of unigenes	99,689	171,127	83,580	42863
Mean length of unigenes (nt)	684		612	

Reads are assembled using Trinity. The longest assembled sequences are called contigs. Sequences without Ns and cannot be extended on either end are defined as unigenes.

**Table 2:** *D. nobile* unigenes that share homology with cytokinin-associated genes in *Arabidopsis.*

Funtion	Unigene ID	Unigene length (bp)	Homolog gene
Cytokinin synthesis	Unigene16780_TRA	1536	*AtIPT9*
	Unigene21318_TRA	375	*AtIPT5*
	Unigene494_TRA	829	*AtLOG8*
	CL1164.Contig1_TRA	310	*AtCYP735A1*
Cytokinin metabolism	Unigene45836_TRA	412	*AtCKX2*
	CL4305.Contig3_TRA	1795	*AtCKX1*
	CL15712.Contig1_TRA	1741	*AtCKX7*
	CL9972.Contig1_TRA	1391	*AtCKX3*
	CL9972.Contig2_TRA	1366	*AtCKX3*
	CL9972.Contig3_TRA	775	*AtCKX3*
	CL9972.Contig4_TRA	750	*AtCKX5*
Cytokinin receptor	CL1514.Contig4_TRA	4493	*AtAHK3*
Cytokinin signal transduction	Unigene4754_TRA	1291	*AtARR9*
	CL9210.Contig1_TRA	960	*AtARR9*
	Unigene33877_TRA	678	*AtARR18*
	CL17707.Contig1_TRA	1084	*AtARR11*

**Table 3:** Differential expression of MADS-box genes in three DGE libraries.

Gene ID	Homologs	CK-RPKM	LT-RPKM	TDZ-RPKM
Unigene28095_TRA	DtpsMADS1 [Doritaenopsis hybrid cultivar] (AP1-like)	17.20851	20.68192	11.42396
CL12116.Contig1_TRA*^2^	DtpsMADS1 [Doritaenopsis hybrid cultivar] (AP1/FUL)	0.819994	0.835173	0
CL12116.Contig2_TRA*^3^	DnMADS1 [D. nobile] (AP1/FUL)	0	0.287894	0.284313
CL12116.Contig3_TRA*^3^	DnMADS1 [D. nobile] (AP1/FUL)	1.035041	0	0
CL15749.Contig1_TRA*^2^	DthylFL2 [D. thyrsiflorum] (AP1/FUL)	0	0	0.2565
CL15749.Contig2_TRA*^1^	DthylFL2 [D. thyrsiflorum] (AP1/FUL)	0.289641	0.590005	0.291333
CL1911.Contig1_TRA*^3^	DthylFL2 [D. thyrsiflorum] (AP1/FUL)	1.889471	2.405559	0.950255
CL16053.Contig1_TRA	DthylFL3 [D. thyrsiflorum] (AP1/FUL)	5.326266	8.266453	3.061361
Unigene31055_TRA	CfAP11 [Cymbidium faberi] (AP1/FUL)	8.965962	9.740725	5.411004
CL16053.Contig2_TRA*^1^	CfAP11 [Cymbidium faberi] (AP1-like)	0.347569	0.708006	0.3496
CL16305.Contig1_TRA	AP1-like MADS-box protein [Cymbidium ensifolium]	135.6404	228.2286	160.6501
Unigene24218_TRA	ORAP13 [Phalaenopsisamabilis] (AP1-like)	1.501192	2.117049	2.090716
Unigene24219_TRA	ORAP13 [Phalaenopsisamabilis] (AP1-like)	2.98443	5.572736	2.12632
CL4716.Contig1_TRA	PeSOC1 [Phalaenopsisequestris]	7.89774	7.097589	10.98124
Unigene29514_TRA	PeSOC1 [Phalaenopsisequestris]	130.3432	144.6503	185.2895
Unigene34271_TRA	PeSOC1 [Phalaenopsisequestris]	22.49251	17.94527	30.1652
Unigene44320_TRA*^2^	HvSOC1 [Hordeumvulgare]	1.232624	1.25544	0.49593
Unigene44321_TRA*^1^	HvSOC1 [Hordeumvulgare]	0.324345	0.660697	0.326239
CL14402.Contig1_TRA	StMADS11-1 [Elaeisguineensis] (SVP-like)	70.08733	85.46903	59.67854
CL14402.Contig2_TRA	AtqMADS5 [Agave tequilana] (SVP-like)	1.823389	1.857141	2.200849
CL12576.Contig1_TRA*^3^	DOMADS3 [D.grex Madame Thong-In] (SEP)	1.129999	2.137416	2.273201
CL12576.Contig2_TRA*^3^	DOMADS3 [D.grex Madame Thong-In] (SEP)	0.396523	0	0
Unigene50333_TRA*^1^	DcOAG1 [D.crumenatum] (AG-like)	1.711436	0.871558	1.506255
Unigene54214_TRA	DnMADS2 [D. nobile](AG-like)	2.003781	2.040872	2.015487
CL3879.Contig1_TRA*^1^	OsMADS27 [Oryza sativa] (AGL16)	2.426993	0.463485	1.983452
CL3879.Contig2_TRA	OsMADS27 [Oryza sativa] (AGL21)	1.779795	2.966303	2.766661
Unigene27314_TRA	CsatAGL6a [Crocus sativus] (AGL6)	10.54833	7.594603	11.15874
CL7312.Contig2_TRA*^2^	mads box protein [Ricinuscommunis] (ZMM17)	0	0	0.1311

Expression differences were determined by RPKM (Reads Per kb per Million reads) values calculated according to the method provided by Mortazavi and Williams (2008). Genes with a log2Ratio ≥ 1 of CK and LT samples were marked with *1, those of CK and TDZ samples were marked with *2, and genes with a log2Ratio ≥ 1 both of CK and LT and of CK and TDZ were marked with *3.

## References

[b1] Sung S. & Amasino R. M. Vernalization in *Arabidopsis thaliana* is mediated by the PHD finger protein VIN3. Nature 427(6970), 159–164 (2004). 14712276 10.1038/nature02195

[b2] Gendall A. R., Levy Y. Y., Wilson A. & Dean C. The *VERNALIZATION 2* gene mediates the epigenetic regulation of vernalization in Arabidopsis. Cell 107(4), 525–535 (2001). 11719192 10.1016/s0092-8674(01)00573-6

[b3] Levy Y. Y., Mesnage S., Mylne J. S., Gendall A. R. & Dean C. Multiple roles of *Arabidopsis VRN1* in vernalization and flowering time control. Science 297(5579), 243–246 (2002). 12114624 10.1126/science.1072147

[b4] Bernier G. My favourite flowering image: the role of cytokinin as a flowering signal. Journal of Experimental Botany, doi: 10.1093/jxb/err114 (2014). 21586428

[b5] Tarkowská D. . Cytokinins in shoot apices of Brassica napus plants during vernalization. Plant Science 187(4), 105–112 (2012). 22404838 10.1016/j.plantsci.2012.02.003

[b6] D’Aloia M. . Cytokinin promotes flowering of Arabidopsis via transcriptional activation of the *FT* paralogue TSF. Plant Journal 65(6), 972–979 (2011). 21205031 10.1111/j.1365-313X.2011.04482.x

[b7] Wong C. E., Singh M. B. & Bhalla P. L. The dynamics of soybean leaf and shoot apical meristem transcriptome undergoing floral initiation process. Plos One 8(6), e65319–e65319 (2013). 23762343 10.1371/journal.pone.0065319 PMC3675103

[b8] Peter B. Systematics of dendrobiinae (orchidaceae), with special reference to australian taxa. Botanical Journal of the Linnean Society 166(2), 105–126 (2011).

[b9] Leonhardt K. W. Potted, blooming dendrobium orchids. Horttechnology 10(3), 431–432 (2000).

[b10] Rotor G. B. Daylength and temperature in relation to growth and flowering of orchids. Cornell Experiment Station Bulletin 885, 3–47 (1952).

[b11] Rotor G. B. The photoperiodic and temperature responses of orchids in The orchids: a scientific survey (ed. Withner C. L.) 397–416 (Ronald Press, 1959).

[b12] GOH C. J. Hormonal regulation of flowering in a sympodial orchid hybrid *Dendrobium* louisae. New Phytologist 82(2), 375–380 (2006).

[b13] Wen Z., Liu Y., Lin S., Lin Y. & Liu W. Cloning and expression analysis of a *vrn1*-like gene from *Dendrobium nobile*. Journal of South China Normal University 45(4), 109–114 (2013).

[b14] Grabherr M. G. . Full-length transcriptome assembly from RNA-seq data without a reference genome. Nature Biotechnology 29(7), 644–652 (2011). 10.1038/nbt.1883 PMC3571712 21572440

[b15] He Y. Control of the transition to flowering by chromatin modifications. Molecular Plant 2(4), 554–564 (2009). 19825638 10.1093/mp/ssp005

[b16] Shannon S. & Meeks-Wagner D. R. A mutation in the Arabidopsis *TFL1* gene affects inflorescence meristem development. Plant Cell 3(9), 877–892 (1991). 12324621 10.1105/tpc.3.9.877 PMC160057

[b17] Alexandre C. M. & Hennig L. FLC or not FLC: the other side of vernalization. Journal of Experimental Botany. 59(6), 1127–1135 (2008). 18390846 10.1093/jxb/ern070

[b18] Kim D. H., Doyle M. R., Sung S. & Amasino R. M. Vernalization: winter and the timing of flowering in plants. Annual Review of Cell and Developmental Biology 25, 277–299 (2009). 10.1146/annurev.cellbio.042308.113411 19575660

[b19] Liang S. . Transcriptional regulations on the low-temperature-induced floral transition in an *orchidaceae* species, *Dendrobium nobile*: an expressed sequence tags analysis. Comparative & Functional Genomics 2012(1), 757–801 (2012). 10.1155/2012/757801 PMC3328899 22550428

[b20] Putterill J., Robson F., Lee K., Simon R. & Coupland G. The *CONSTANS* gene of *Arabidopsis* promotes flowering and encodes a protein showing similarities to zinc finger transcription factors. Cell 80(6), 847–57 (1995). 7697715 10.1016/0092-8674(95)90288-0

[b21] Mcgaw B. A. Cytokinin Biosynthesis and Metabolism in Plant Hormones and their Role in Plant Growth and Development (ed. Davies P. J.) 24–42 (M.Nijhoff, 1987).

[b22] Zürcher E. & Müller B. Cytokinin synthesis, signaling, and function—advances and new insights. International Review of Cell & Molecular Biology 324, 1–38 (2016). 27017005 10.1016/bs.ircmb.2016.01.001

[b23] Audic S. & Claverie J. M. The significance of digital gene expression profiles. GenomeResearch 7(10), 986–95 (1997). 10.1101/gr.7.10.986 9331369

[b24] Boggio R. & Chiocca S. Gam1 and the SUMO pathway. Cell Cycle 4(4), 533–5 (2005). 15876861 10.4161/cc.4.4.1605

[b25] Wahl V. . Regulation of flowering by trehalose-6-phosphate signaling in *Arabidopsis thaliana*. Science 339(6120), 704–7 (2013). 23393265 10.1126/science.1230406

[b26] Silva J. A. T. D., Aceto S., Liu W., Yu H. & Kanno A. Genetic control of flower development, color and senescence of *Dendrobium* orchids. Scientia Horticulturae 175(1), 74–86 (2014).

[b27] Cai J. . The genome sequence of the orchid *phalaenopsis equestris*. Nature Genetics 47(1), 65–72 (2014). 25420146 10.1038/ng.3149

[b28] Yan L. . The genome of *Dendrobium officinale* illuminates the biology of the important traditional Chinese orchid herb. Molecular Plant 8, 922–934 (2014). 25825286 10.1016/j.molp.2014.12.011

[b29] Chang Y. Y. . Characterization of *Oncidium* “gower ramsey” transcriptomes using 454 GS-FLX pyrosequencing and their application to the identification of genes associated with flowering time. Plant & Cell Physiology 52(9), 1532–45 (2011). 21785129 10.1093/pcp/pcr101

[b30] Zhang J. . Transcriptome analysis of cymbidium sinense and its application to the identification of genes associated with floral development. BMC Genomics 14(1), 1–17 (2013). 23617896 10.1186/1471-2164-14-279 PMC3639151

[b31] Su C. L. . Orchidstra: an integrated orchid functional genomics database. Plant & Cell Physiology 54(2), e11–e11(2013). 23324169 10.1093/pcp/pct004 PMC3583029

[b32] Yang W. . Regulation of meristem morphogenesis by cell wall synthases in arabidopsis. Current Biology Cb 26(11), 1404 (2016). 27212401 10.1016/j.cub.2016.04.026 PMC5024349

[b33] Kushwah S. & Laxmi A. The interaction between glucose and cytokinin signal transduction pathway in *Arabidopsis thaliana*. Plant Cell & Environment 37(1), 235–253 (2014). 10.1111/pce.12149 23763631

[b34] Krucoff M. . Florigenic effect of gibberellin on flowering according to period of chilling treatment in *lavandula*×*intermedia*. Horticulture Journal 85(2), 169–176 (2015).

[b35] Li M. Z. . DELLA proteins interact with FLC to repress flowering transition. Journal of Integrative Plant Biology 58(7), 642–655 (2016). 26584710 10.1111/jipb.12451

[b36] Yamaguchi N. . Gibberellin acts positively then negatively to control onset of flower formation in *Arabidopsis*. Science 344(6184), 638–641 (2014). 24812402 10.1126/science.1250498

[b37] Moon J. . The *SOC1* mads‐box gene integrates vernalization and gibberellin signals for flowering in *Arabidopsis*. Plant Journal 35(5), 613–23 (2003). 12940954 10.1046/j.1365-313x.2003.01833.x

[b38] Han Y., Zhang C., Yang H. & Jiao Y. Cytokinin pathway mediates apetala1 function in the establishment of determinate floral meristems In Arabidopsis. PNAS 111(18), 6840–6845 (2014). 24753595 10.1073/pnas.1318532111 PMC4020066

[b39] Mortazavi A., Williams B. A., Mccue K., Schaeffer L. & Wold B. Mapping and quantifying mammalian transcriptomes by RNA-seq. Nature Methods 5(7), 621–628 (2008). 18516045 10.1038/nmeth.1226 PMC13303166

[b40] Sturn A., Quackenbush J. & Trajanoski Z. Genesis: cluster analysis of microarray data. Bioinformatics 18(1), 207–208 (2002). 11836235 10.1093/bioinformatics/18.1.207

[b41] Clamp M., Cuff J., Searle S. M. & Barton G. J. The jalview java alignment editor. Bioinformatics 20(3), 426–427 (2004). 14960472 10.1093/bioinformatics/btg430

