# Supplementary material for: Comparative Transcriptomic Analysis of Vernalization- and Cytokinin-Induced Floral Transition in Dendrobium nobile
Source: Sci Rep. 2017 Mar 31;7:45748. doi: 10.1038/srep45748 (PMC5374638; doi:10.1038/srep45748)
Supplement: Supplementary Information [file srep45748-s1.doc]

**Comparative Transcriptomic Analysis of Vernalization- and Cytokinin-Induced Floral Transition in *Dendrobium nobile* - Supplementary Information**

Zhenzhen Wen^1,2^, Wenzhong Guo^1^, Jinchi Li^1^, Haisheng Lin^1^,Chunmei He^1^,Yunquan Liu^1^, Qunyu Zhang^1,*2^, Wei Liu^1,*1^

1. South China Agricultural University, College of life sciences, Guangzhou, 510642, China

2. Guangdong Engineering Polytechnic, Guangzhou, 510520, China

*^1^Corresponding Author1, Email: [liuwei@scau.edu.cn](mailto:liuwei@scau.edu.cn), Tax:(+86)020-85282180

*^2^Corresponding Author 2, Email: zqy[@scau.edu.cn](mailto:liuwei@scau.edu.cn), Tax:(+86)020-85282180

Table S1 Primers used for RT-qPCR

| **Gene name** | **Primers** |
| --- | --- |
| DnActin | 5’-TGCTTCTAAAGTTTGCTAT-3’  5’-ACTCAATCCAGACTACAA-3’ |
| Unigene494_TRA | 5’-TCGAGCCGGTGTTTAGTGATGC-3’  5’-TCGAGCCGTGTTTAGTGATGC-3’ |
| CL12116.Contig3_TRA | 5’-GGCTGATTGGCACCTTGAA-3’  5’-CATTGGCTCTTGCTTGAAAC-3’ |
| CL17034.Contig1_TRA | 5’-GGCAAGGGAGGCAGAGACGACGAG-3’  5’-GGGGACCGGAAGAGCTGGAAACT-3’ |
| Unigene11479_TRA | 5’-ATGCGGGAGGAGGAGGTGGACGAT-3’  5’-CGCCGGCGGTTGGAGGAAAAAT-3’ |
| CL15581.Contig2_TRA | 5’-AGGATGGCTCGGTCTGG-3’  5’-CAATTGGGAACGCACATAG-3’ |
| CL16305.Contig1_TRA | 5’-CCGAGGTGGCGCTTATCATCTTC-3’  5’-GTTTCCGCTTTGTATCCTGTGTCA-3’ |
| Unigene43297_TRA | 5’-GCGGCCCGGCGACCACTC-3’  5’-GCCGCCAAAACCCCCTACACCAT-3’ |
| Unigene27426_TRA | 5’-AATAGTCCAACACAGTATA-3’ 5’-CAGCAAAGCAATATAATAAC-3’ |
| Unigene35524_TRA | 5’-GGCAGCGTATGAAGAACAAA-3’  5’-AGCGAAGAACAGCGAGGTAG-3’ |
| Unigene1880_TRA | 5’-TTCACAGCAACAACCGAGAT-3’  5’-AACGGCATTAGCACCTTCAT-3’ |
| CL2388.Contig3_TRA | 5’-CTTTCTTTGCCGTCCCATAA-3’  5’-GTATCCATTCACCGCCTCTG-3’ |
| Unigene24590_TRA | 5’-ACATGCGGTGAATGGATAAA-3’  5’-GGTGATTAGGTGCGTGAAGTC-3’ |
| Unigene3219_TRA | 5’-TTATCCATCCAGGCTTAGGG-3’  5’-ACTTGCTTTGCCCACTGAAA-3’ |
| Unigene39396_TRA | 5’-AGCAGTAATGGCGAGAAGGA-3’  5’-TGCCCACCAAAGAAGAAAGT-3’ |
| CL5968.Contig1_TRA | 5’-GGGACTTACGGCAATCTTCA-3’  5’-AAGCGATACCAGGCTCCATA-3’ |
| CL3148.Contig1_TRA | 5’-TTTCGGTTCTCATTCGTTGA-3’  5’-TGTGCTGTCCTGCTTCTGAC-3’ |
| Unigene45836_TRA | 5’-TTTGGAGCATTTGGAGAACC-3’  5’-TTGCTTTGGGATCATATCTGC-3’ |
| CL12022.Contig1_TRA | 5’-ACCCACCTTTCCTCATCCTC-3’  5’-CTTGCTCACAGAATCCACCA-3’ |
| CL7022.Contig1_TRA | 5’-ACGCTTCTGTTGTTGGCTCT-3’  5’-CCACACCCATCCCATCTTTA-3’ |
| CL6189.Contig2_TRA | 5’-CTGTAAGGCGAGGAAGAAGG-3’  5’-ACGACCAACTAAGGCTCTGTG-3’ |

**T**able S2 Biological pathway assigned by KEGG

| **Pathway** | **Number of unigenes** | **Percentage of unigenes** |
| --- | --- | --- |
| [Metabolic pathways](../../../../F:%5C%E9%97%BB%E7%9C%9F%E7%8F%8D%5C%E5%AD%A6%E7%94%9F%E5%AE%9E%E9%AA%8C%5C%E9%83%AD%E6%96%87%E4%B8%AD%5C%E8%BD%AC%E5%BD%95%E7%BB%84%5C%E8%BD%AC%E5%BD%95%E7%BB%84_30371%5C%E8%BD%AC%E5%BD%95%E7%BB%84%5Cannotation%5CKEGG%5CTRA-Unigene.fa.htm#gene1) | 7318 | 24.27% |
| [Biosynthesis of secondary metabolites](../../../../F:%5C%E9%97%BB%E7%9C%9F%E7%8F%8D%5C%E5%AD%A6%E7%94%9F%E5%AE%9E%E9%AA%8C%5C%E9%83%AD%E6%96%87%E4%B8%AD%5C%E8%BD%AC%E5%BD%95%E7%BB%84%5C%E8%BD%AC%E5%BD%95%E7%BB%84_30371%5C%E8%BD%AC%E5%BD%95%E7%BB%84%5Cannotation%5CKEGG%5CTRA-Unigene.fa.htm#gene2) | 3292 | 10.92% |
| [Plant-pathogen interaction](../../../../F:%5C%E9%97%BB%E7%9C%9F%E7%8F%8D%5C%E5%AD%A6%E7%94%9F%E5%AE%9E%E9%AA%8C%5C%E9%83%AD%E6%96%87%E4%B8%AD%5C%E8%BD%AC%E5%BD%95%E7%BB%84%5C%E8%BD%AC%E5%BD%95%E7%BB%84_30371%5C%E8%BD%AC%E5%BD%95%E7%BB%84%5Cannotation%5CKEGG%5CTRA-Unigene.fa.htm#gene3) | 1560 | 5.17% |
| [Plant hormone signal transduction](../../../../F:%5C%E9%97%BB%E7%9C%9F%E7%8F%8D%5C%E5%AD%A6%E7%94%9F%E5%AE%9E%E9%AA%8C%5C%E9%83%AD%E6%96%87%E4%B8%AD%5C%E8%BD%AC%E5%BD%95%E7%BB%84%5C%E8%BD%AC%E5%BD%95%E7%BB%84_30371%5C%E8%BD%AC%E5%BD%95%E7%BB%84%5Cannotation%5CKEGG%5CTRA-Unigene.fa.htm#gene4) | 1305 | 4.33% |
| [Endocytosis](../../../../F:%5C%E9%97%BB%E7%9C%9F%E7%8F%8D%5C%E5%AD%A6%E7%94%9F%E5%AE%9E%E9%AA%8C%5C%E9%83%AD%E6%96%87%E4%B8%AD%5C%E8%BD%AC%E5%BD%95%E7%BB%84%5C%E8%BD%AC%E5%BD%95%E7%BB%84_30371%5C%E8%BD%AC%E5%BD%95%E7%BB%84%5Cannotation%5CKEGG%5CTRA-Unigene.fa.htm#gene5) | 1288 | 4.27% |
| [Glycerophospholipid metabolism](../../../../F:%5C%E9%97%BB%E7%9C%9F%E7%8F%8D%5C%E5%AD%A6%E7%94%9F%E5%AE%9E%E9%AA%8C%5C%E9%83%AD%E6%96%87%E4%B8%AD%5C%E8%BD%AC%E5%BD%95%E7%BB%84%5C%E8%BD%AC%E5%BD%95%E7%BB%84_30371%5C%E8%BD%AC%E5%BD%95%E7%BB%84%5Cannotation%5CKEGG%5CTRA-Unigene.fa.htm#gene6) | 1222 | 4.05% |
| [Spliceosome](../../../../F:%5C%E9%97%BB%E7%9C%9F%E7%8F%8D%5C%E5%AD%A6%E7%94%9F%E5%AE%9E%E9%AA%8C%5C%E9%83%AD%E6%96%87%E4%B8%AD%5C%E8%BD%AC%E5%BD%95%E7%BB%84%5C%E8%BD%AC%E5%BD%95%E7%BB%84_30371%5C%E8%BD%AC%E5%BD%95%E7%BB%84%5Cannotation%5CKEGG%5CTRA-Unigene.fa.htm#gene7) | 1206 | 4% |
| [RNA transport](../../../../F:%5C%E9%97%BB%E7%9C%9F%E7%8F%8D%5C%E5%AD%A6%E7%94%9F%E5%AE%9E%E9%AA%8C%5C%E9%83%AD%E6%96%87%E4%B8%AD%5C%E8%BD%AC%E5%BD%95%E7%BB%84%5C%E8%BD%AC%E5%BD%95%E7%BB%84_30371%5C%E8%BD%AC%E5%BD%95%E7%BB%84%5Cannotation%5CKEGG%5CTRA-Unigene.fa.htm#gene8) | 1197 | 3.97% |
| [Ether lipid metabolism](../../../../F:%5C%E9%97%BB%E7%9C%9F%E7%8F%8D%5C%E5%AD%A6%E7%94%9F%E5%AE%9E%E9%AA%8C%5C%E9%83%AD%E6%96%87%E4%B8%AD%5C%E8%BD%AC%E5%BD%95%E7%BB%84%5C%E8%BD%AC%E5%BD%95%E7%BB%84_30371%5C%E8%BD%AC%E5%BD%95%E7%BB%84%5Cannotation%5CKEGG%5CTRA-Unigene.fa.htm#gene9) | 1004 | 3.33% |
| [Ribosome](../../../../F:%5C%E9%97%BB%E7%9C%9F%E7%8F%8D%5C%E5%AD%A6%E7%94%9F%E5%AE%9E%E9%AA%8C%5C%E9%83%AD%E6%96%87%E4%B8%AD%5C%E8%BD%AC%E5%BD%95%E7%BB%84%5C%E8%BD%AC%E5%BD%95%E7%BB%84_30371%5C%E8%BD%AC%E5%BD%95%E7%BB%84%5Cannotation%5CKEGG%5CTRA-Unigene.fa.htm#gene10) | 899 | 2.98% |
| [Ubiquitin mediated proteolysis](../../../../F:%5C%E9%97%BB%E7%9C%9F%E7%8F%8D%5C%E5%AD%A6%E7%94%9F%E5%AE%9E%E9%AA%8C%5C%E9%83%AD%E6%96%87%E4%B8%AD%5C%E8%BD%AC%E5%BD%95%E7%BB%84%5C%E8%BD%AC%E5%BD%95%E7%BB%84_30371%5C%E8%BD%AC%E5%BD%95%E7%BB%84%5Cannotation%5CKEGG%5CTRA-Unigene.fa.htm#gene19) | 505 | 1.67% |
| [Zeatin biosynthesis](../../../../F:%5C%E9%97%BB%E7%9C%9F%E7%8F%8D%5C%E5%AD%A6%E7%94%9F%E5%AE%9E%E9%AA%8C%5C%E9%83%AD%E6%96%87%E4%B8%AD%5C%E8%BD%AC%E5%BD%95%E7%BB%84%5C%E8%BD%AC%E5%BD%95%E7%BB%84_30371%5C%E8%BD%AC%E5%BD%95%E7%BB%84%5Cannotation%5CKEGG%5CTRA-Unigene.fa.htm#gene28) | 336 | 1.11% |
| [Circadian rhythm - plant](../../../../F:%5C%E9%97%BB%E7%9C%9F%E7%8F%8D%5C%E5%AD%A6%E7%94%9F%E5%AE%9E%E9%AA%8C%5C%E9%83%AD%E6%96%87%E4%B8%AD%5C%E8%BD%AC%E5%BD%95%E7%BB%84%5C%E8%BD%AC%E5%BD%95%E7%BB%84_30371%5C%E8%BD%AC%E5%BD%95%E7%BB%84%5Cannotation%5CKEGG%5CTRA-Unigene.fa.htm#gene44) | 215 | 0.71% |
| [Basal transcription factors](../../../../F:%5C%E9%97%BB%E7%9C%9F%E7%8F%8D%5C%E5%AD%A6%E7%94%9F%E5%AE%9E%E9%AA%8C%5C%E9%83%AD%E6%96%87%E4%B8%AD%5C%E8%BD%AC%E5%BD%95%E7%BB%84%5C%E8%BD%AC%E5%BD%95%E7%BB%84_30371%5C%E8%BD%AC%E5%BD%95%E7%BB%84%5Cannotation%5CKEGG%5CTRA-Unigene.fa.htm#gene62) | 165 | 0.55% |

**Table S3. *D. nobile* unigenes that share homology with flowering time genes in *Arabidopsis***

| **Pathway** | **Homolog gene** | **Function** | **ArabidopsisMIPS code** | **Unigene ID** | **Unigene length (bp)** | **E-value** |
| --- | --- | --- | --- | --- | --- | --- |
| Photoperiodic | *CO* | Activator | AT5G15480 | CL4430.Contig2_TRA  CL5412.Contig2_TRA | 1422  801 | 5e-55  1e-55 |
|  | *COP1* | Repressor | AT2G32950 | CL4710.Contig1_TRA  CL4710.Contig2_TRA  CL4710.Contig3_TRA | 2844  2402  2102 | 4e-12  3e-12  8e-12 |
|  | *CRY1* | Activator | AT4G08920 | Unigene48360_TRA  CL13673.Contig2_TRA  CL9807.Contig2_TRA | 858  745  709 | 0  6e-106  3e-80 |
|  | *CRY2* | Activator | AT1G04400 | Unigene35211_TRA  Unigene35213_TRA | 711  1653 | 3e-143  1e-146 |
|  | *FD* | Activator | AT4G35900 | Unigene31059_TRA | 732 | 8e-12 |
|  | *FKF1* | Activator | AT1G68050 | CL16409.Contig1_TRA | 2068 | 0 |
|  | *GI* | Activator | AT1G22770 | Unigene32983_TRA  CL6541.Contig2_TRA | 1087  3316 | 2e-77  0 |
|  | *SPA* | Repressor | AT2G46340 | CL10740.Contig2_TRA  CL5078.Contig2_TRA | 347  385 | 1e-17  3e-33 |
|  | *PHYA* | Activator | AT1G09570 | CL11150.Contig2_TRA  Unigene32223_TRA  Unigene15017_TRA | 291  217  293 | 1e-27  9e-22  4e-25 |
|  | *PHYB* | Repressor | AT2G18790 | Unigene39212_TRA  Unigene46899_TRA | 2749  2283 | 0  0 |
| Vernalization | *FRI* | Repressor | AT4G00650 | Unigene3986_TRA | 1473 | 4e-11 |
|  | *LHP1* | Activator | AT5G17690 | CL12480.Contig1_TRA  CL12480.Contig2_TRA | 1700  1614 | 3e-18  2e-18 |
|  | *VIN3* | Activator | AT5G57380 | CL7054.Contig1_TRA  CL17269.Contig1_TRA  Unigene2487_TRA | 2681  2452  1207 | 4e-57  1e-100  2e-48 |
|  | *AGL19* | Activator |  | CL4716.Contig1_TRA | 606 | 4e-09 |
| Autonomous | *FCA* | Activator | AT4G16280 | Unigene43297_TRA  CL1567.Contig1_TRA  CL4665.Contig1_TRA | 1131  1235  1726 | 2e-98  1e-50  2e-96 |
|  | *FLD* | Activator | AT3G10390 | Unigene35537_TRA | 2278 | 0 |
|  | *FPA* | Activator | AT2G43410 | CL18759.Contig2_TRA | 3431 | 1e-156 |

**Table S3. *D. nobile* unigenes that share homology with flowering time genes (continued)**

| **Pathway** | **Homolog gene** | **Function** | **ArabidopsisMIPS code** | **Unigene ID** | **Unigene length (bp)** | **E-value** |
| --- | --- | --- | --- | --- | --- | --- |
|  | *FY* | Activator | AT5G13480 | Unigene33422_TRA | 2466 | 0 |
|  | *LD* | Activator | AT4G02560 | CL2385.Contig2_TRA | 3907 | 7e-131 |
| GA | *GAI* | Repressor | AT1G14920 | Unigene31899_TRA  Unigene34408_TRA  Unigene39593_TRA  CL3444.Contig2_TRA | 1138  985  1507  1169 | 1e-94  2e-110  2e-45  1e-163 |
|  | *GID1* | Activator | AT3G05120 | Unigene33054_TRA  CL15632.Contig2_TRA  CL1158.Contig3_TRA | 2017  2042  1251 | 8e-173  2e-160  3e-74 |
|  | *SPY* | Repressor | AT3G11540 | Unigene42044_TRA | 3506 | 0 |
| Ambient temperature | *ARP6* | Repressor | AT3G33520 | Unigene48973_TRA | 1502 | 0 |
|  | *SVP* | Repressor | AT2G22540 | CL14402.Contig2_TRA  CL14402.Contig2_TRA  CL3181.Contig1_TRA  CL3181.Contig5_TRA  CL3181.Contig7_TRA | 1158  1029  1027  942  861 | 1e-85  4e-86  4e-84  1e-81  9e-83 |
|  | *SPL* | Activator | AT2G47070 | Unigene41543_TRA  CL13733.Contig1_TRA | 1000  3928 | 5e-78  1e-167 |
| Chromatin regulation | *LDL1* | Represses FLC | AT1G62830 | Unigene41333_TRA | 1196 | 2e-170 |
|  | *LDL2* | Represses FLC | AT3G13682 | Unigene3370_TRA  Unigene39133_TRA  Unigene46354_TRA | 960  3166  1444 | 3e-80  1e-84  0 |
|  | *REF6* | Represses FLC | AT3G48430 | Unigene37861_TRA | 1057 | 5e-66 |
|  | *ATX2* | Activates FLC | AT1G05830 | Unigene30327_TRA | 2387 | 0 |
|  | *HUB1* | Activates FLC | AT2G44950 | Unigene26884_TRA | 491 | 8e-50 |
|  | *UBC1* | Activates FLC | AT1G14400 | CL972.Contig2_TRA | 925 | 8e-110 |
|  | *CLF* | Represses FLC/FT | AT2G23380 | CL4669.Contig1_TRA | 864 | 8e-58 |
|  | *FIE* | Represses FLC/FT | AT3G20740 | CL15581.Contig1_TRA | 1328 | 0 |
|  | *EMF2* | Represses FLC/FT | AT5G51230 | CL12672.Contig3_TRA | 2073 | 2e-126 |
|  | *MSI1* | Represses FLC | AT5G58230 | CL1433.Contig2_TRA | 586 | 8e-74 |
|  | *JMJ14* | Represses FT | AT4G20400 | CL2795.Contig1_TRA  CL2795.Contig2_TRA | 2667  2643 | 0  0 |

**Table S3. *D. nobile* unigenes that share homology with flowering time genes (continued)**

| **Pathway** | **Homolog gene** | **Function** | **ArabidopsisMIPS code** | **Unigene ID** | **Unigene length (bp)** | **E-value** |
| --- | --- | --- | --- | --- | --- | --- |
| Floral integrator | *FT* | Activator | AT1G65480 | CL6101.Contig2_TRA  Unigene8516_TRA | 575  213 | 1e-13  1e-24 |
|  | *SOC1* | Activator | AT2G45660 | Unigene29514_TRA  Unigene34271_TRA | 1121  751 | 1e-66  3e-58 |
|  | *LFY* | Activator | AT5G61850 | Unigene11479_TRA | 214 | 2e-21 |
|  | *TFL1* | Repressor | AT5G03840 | Unigene16756_TRA | 470 | 1e-77 |

**Table S4. *CONSTANS*-like unigenes identified in *D. nobile***

| **GeneID** | **Homologs [species]** | **E-value** |
| --- | --- | --- |
| CL1918.Contig1_TRA | AtCOL2 | 2.00E-10 |
| CL1918.Contig2_TRA | AtCOL2 | 2.00E-10 |
| CL1918.Contig3_TRA | AtCOL2 | 2.00E-10 |
| CL1918.Contig4_TRA | AtCOL2 | 2.00E-10 |
| CL1918.Contig5_TRA | AtCOL2 | 2.00E-10 |
| CL1918.Contig6_TRA | AtCOL2 | 1.00E-10 |
| CL198.Contig1_TRA | AtCOL2 | 3.00E-07 |
| CL198.Contig2_TRA | AtCOL2 | 5.00E-07 |
| CL198.Contig3_TRA | AtCOL2 | 5.00E-07 |
| CL198.Contig4_TRA | AtCOL2 | 3.00E-07 |
| CL4430.Contig1_TRA | AtCOL13 | 2.00E-54 |
| CL4430.Contig2_TRA | AtCOL13 | 2.00E-54 |
| CL5412.Contig1_TRA | CONSTANS-like protein [*D. loddigesii*] | 1E-122 |
| CL5412.Contig2_TRA | CONSTANS-like protein [*D. loddigesii*] | 7.00E-42 |
| CL5412.Contig3_TRA | CONSTANS-like protein [*D. loddigesii*] | 0 |
| CL80.Contig1_TRA | COL10 [*Vitis vinifera*] | 4E-117 |
| CL80.Contig2_TRA | AtCOL10 | 8.00E-75 |
| CL80.Contig3_TRA | AtCOL9 | 1E-104 |
| CL8950.Contig1_TRA | COL14[*Malus* x domestica] | 1.00E-13 |
| CL8950.Contig2_TRA | COL14[*Malus* x domestica] | 5.00E-13 |
| Unigene13700_TRA | AtCOL5 | 9.00E-09 |
| Unigene17367_TRA | CONSTANS-like 1 [*P. amabilis*] | 2.00E-25 |
| Unigene28586_TRA | AtCOL4 | 3.00E-17 |
| Unigene3016_TRA | COL16 [*Vitis vinifera*] | 1.00E-30 |
| Unigene33423_TRA | CONSTANS-like 1 [*P. amabilis*] | 3.00E-16 |
| Unigene33424_TRA | CONSTANS-like 1 [*P. amabilis*] | 5.00E-32 |
| Unigene34158_TRA | AtCOL15 | 1.00E-42 |
| Unigene35845_TRA | AtCOL2 | 2.00E-10 |
| Unigene39656_TRA | AtCOL16 | 7.00E-29 |
| Unigene39804_TRA | AtCOL2 | 2.00E-12 |
| Unigene41139_TRA | COL16 [*Vitis vinifera*] | 2.00E-86 |
| Unigene919_TRA | COL13 [*Brachypodium distachyon*] | 3.00E-13 |

**Table S5. MADS-box unigenes identified in *D. nobile***

| **GeneID** | **Homologs [species]** | **E-value** |
| --- | --- | --- |
| CL12116.Contig1_TRA | *MADS1* [x *Doritaenopsis* hybrid cultivar] | 2.00E-64 |
| CL12116.Contig2_TRA | *DnMADS1* [*D. nobile*] | 1.00E-85 |
| CL12116.Contig3_TRA | *DnMADS1* [*D. nobile*] | 1.00E-31 |
| CL12576.Contig1_TRA | *DOMADS3* [*D.*grex Madame Thong-In] | 8E-111 |
| CL12576.Contig2_TRA | *DOMADS3* [*D.*grex Madame Thong-In] | 1.00E-74 |
| CL14402.Contig1_TRA | MADS box transcription factor [*Elaeis guineensis*] | 1.00E-79 |
| CL14402.Contig2_TRA | MADS box protein 5, partial [*Agave tequilana*] | 2.00E-83 |
| CL14402.Contig3_TRA | MADS box protein 5, partial [*Agave tequilana*] | 1.00E-83 |
| CL15749.Contig1_TRA | *DnMADS2* [*D. nobile*] | 9.00E-13 |
| CL15749.Contig2_TRA | *DnMADS2* [*D. nobile*] | 1.00E-16 |
| CL16053.Contig1_TRA | FRUITFULL-like MADS box protein 3 [*D. thyrsiflorum*] | 1.00E-37 |
| CL16053.Contig2_TRA | AP1-like protein [*C. faber*i] | 7.00E-34 |
| CL16305.Contig1_TRA | AP1-like MADS-box protein [*C. ensifolium*] | 1E-114 |
| CL1911.Contig1_TRA | FRUITFULL-like MADS box protein 2 [*D. thyrsiflorum*] | 3.00E-16 |
| CL3181.Contig1_TRA | PREDICTED: MADS-box protein SVP [*Vitis vinifera*] | 4.00E-64 |
| CL3181.Contig2_TRA | MADS transcription factor [*Zea mays*] | 1.00E-27 |
| CL3181.Contig3_TRA | MADS transcription factor [*Zea mays*] | 3.00E-23 |
| CL3181.Contig4_TRA | PREDICTED: MADS-box protein SVP [*Vitis vinifera*] | 3.00E-67 |
| CL3181.Contig5_TRA | MADS box transcription factor [*Elaeis guineensis*] | 2.00E-62 |
| CL3181.Contig6_TRA | MADS transcription factor [*Zea mays*] | 2.00E-26 |
| CL3181.Contig7_TRA | MADS box transcription factor [*Elaeis guineensis*] | 1.00E-65 |
| CL3879.Contig1_TRA | predicted protein [*Hordeum vulgaresubsp. vulgare*] | 1.00E-71 |
| CL3879.Contig2_TRA | predicted protein [*Hordeum vulgaresubsp. vulgare*] | 3.00E-35 |
| CL3919.Contig10_TRA | forever young flower protein [*O.*Gower Ramsey] | 2.00E-49 |
| CL3919.Contig11_TRA | *SOC1* [*P. equestris*] | 7.00E-53 |
| CL3919.Contig12_TRA | forever young flower protein [*O. Gower Ramsey*] | 2.00E-23 |
| CL3919.Contig13_TRA | *SOC1* [*P. equestris*] | 6.00E-58 |
| CL3919.Contig14_TRA | *AGL20*-like [*Elaeis guineensis*] | 5.00E-28 |
| CL3919.Contig15_TRA | forever young flower protein [Oncidium Gower Ramsey] | 8.00E-32 |
| CL3919.Contig1_TRA | *SOC1* [*P. equestris*] | 3.00E-32 |
| CL3919.Contig2_TRA | *SOC1* [*P. equestris*] | 2.00E-27 |
| CL3919.Contig3_TRA | *SOC1* [*P. equestris*] | 1.00E-22 |
| CL3919.Contig4_TRA | *AGL20*-like [*Elaeis guineensis*] | 5.00E-17 |
| CL3919.Contig5_TRA | *AGL20*-like [*Elaeis guineensis*] | 5.00E-22 |
| CL3919.Contig6_TRA | *AGL20*-like [*Elaeis guineensis*] | 2.00E-11 |
| CL3919.Contig7_TRA | *AGL20*-like [*Elaeis guineensis*] | 2.00E-25 |
| CL3919.Contig8_TRA | *AGL20*-like [*Elaeis guineensis*] | 1.00E-16 |
| CL3919.Contig9_TRA | *AGL20*-like [*Elaeis guineensis*] | 1.00E-19 |
| CL4716.Contig1_TRA | *SOC1* [*P. equestris*] | 2.00E-16 |
| CL4716.Contig2_TRA | *SOC1* [*P. equestris*] | 4.00E-12 |
| CL7312.Contig1_TRA | MADS1 protein [*Eschscholzia californica*] | 2.00E-88 |
| CL7312.Contig2_TRA | mads box protein [*Ricinus communis*] | 1.00E-35 |
| Unigene14895_TRA | *AGAMOUS*-like transcription factor [*D. crumenatum*] | 4.00E-81 |
| Unigene24218_TRA | AP1-related protein [*P. amabilis*] | 2.00E-95 |
| Unigene24219_TRA | AP1-related protein [*P. amabilis*] | 8.00E-99 |
| Unigene27314_TRA | *AGL6a* [*Crocus sativus*] | 1.00E-39 |
| Unigene28095_TRA | *MADS1* [x *Doritaenopsis* hybrid cultivar] | 1.00E-07 |
| Unigene29514_TRA | *SOC1* [*P. equestris*] | 1.00E-99 |
| Unigene31055_TRA | AP1-like protein [*C. faberi*] | 6.00E-50 |
| Unigene33249_TRA | MADS box protein [*Cichorium intybus*] | 7.00E-08 |
| Unigene33250_TRA | *MADS10* [*Lolium perenne*] | 1.00E-24 |
| Unigene34271_TRA | *SOC1* [*P. equestris*] | 1.00E-51 |
| Unigene40684_TRA | PISTILLATA-like (PI) [Navarretia saximontana] | 1.00E-06 |
| Unigene44320_TRA | HvSOC1 [Hordeum vulgare] |  |
| Unigene44321_TRA  Unigene50333_TRA  Unigene54214_TRA | HvSOC1 [Hordeum vulgare]  DcOAG1 [Dendrobium crumenatum]  DnMADS2 [Dendrobium nobile] |  |

Table S6 Summary statistics of DGE sequencing

| **Summary** | **CK** | **LT** | **TDZ** |
| --- | --- | --- | --- |
| Total reads | 12,226,564 | 11,725,725 | 11,927,226 |
| Total base pairs | 599,101,636 | 574,531,125 | 584,434,074 |
| Only adaptor reads (%) | 120,030 (0.97) | 142,901 (1.20) | 182,809 (1.50) |
| Containing N reads (%) | 34,618 (0.28) | 34,781 (0.29) | 34,374 (0.28) |
| Low quality reads (%) | 17,190 (0.14) | 14,998 (0.13) | 17,979 (0.15) |
| Total clean reads (%) | 12,226,564 (98.61) | 11,725,125 (98.38) | 11,927,226 (98.07) |
| Total mapped reads to gene (%) | 10166231 (83.15) | 9792658 (83.52) | 10112626 (84.79) |
| Perfect match (%) | 8036983 (65.73) | 7809329 (66.60) | 7990018 (66.99) |
| ≤2bp mismatch (%) | 2129248 (17.41) | 1983329 (16.92) | 2122608 (17.80) |
| Unique match (%) | 7104008 (58.10) | 6974897 (59.49) | 7062749 (59.22) |
| Total unmapped reads (%) | 2060333 (16.85) | 1932467 (16.48) | 1814600 (15.21) |

**Table S7 Genes specifically expressed in CK, LT** and TDZ

|  | **UnigeneID** | **RPKM** | **Annotation** | **E-value** |
| --- | --- | --- | --- | --- |
| Unigenes specifically expressed in CK | Unigene49361_TRA | 7.272024 | ABC-type dipeptide transport system, periplasmic component | 7.00E-09 |
|  | Unigene26593_TRA | 7.925141 | Coffea canephora subsp. canephora microsatellite DNA, clone 110K17 | 2.00E-06 |
|  | CL10301.Contig2_TRA | 5.541953 | Type I inositol 1,4,5-trisphosphate 5-phosphatase 12 [Arabidopsis thaliana] | 9.00E-12 |
|  | Unigene25784_TRA | 3.161403 | 30S ribosomal protein S18 [Medicago truncatula] | 2.00E-07 |
|  | CL3912.Contig1_TRA | 2.628403 | Glycine-rich cell wall structural protein 1.8 [Phaseolus vulgaris] | 8.00E-10 |
|  | CL976.Contig1_TRA | 1.07097 | Leucine-rich repeat extensin-like protein 5 [Arabidopsis thaliana] | 6.00E-17 |
|  | CL7425.Contig2_TRA | 17.86808 | ---^a^ | --- |
|  | CL10794.Contig1_TRA | 9.479165 | --- | --- |
|  | Unigene49681_TRA | 9.417417 | --- | --- |
| Unigenes specifically expressed in LT | Unigene49374_TRA | 6.76014 | hypothetical protein AURANDRAFT_64234 [Aureococcus anophagefferens] | 7.00E-06 |
|  | Unigene49797_TRA | 6.09328 | hypothetical protein AURANDRAFT_70520 [Aureococcus anophagefferens] | 1.00E-06 |
|  | Unigene15573_TRA | 5.787122 | Late embryogenesis abundant protein 1 (LEA1) [Oryza sativa subsp. japonica] | 3.00E-09 |
|  | Unigene18575_TRA | 5.629763 | hypothetical protein COCSUDRAFT_62329 [Coccomyxa subellipsoidea C-169] | 1.00E-19 |
|  | CL11700.Contig1_TRA | 5.551964 | 14 kDa proline-rich protein DC2.15 [Medicago truncatula] | 1.00E-06 |
|  | Unigene49392_TRA | 5.506716 | Lysine-rich arabinogalactan protein 19 (AGP19) [Arabidopsis thaliana] | 4.00E-08 |
|  | Unigene33927_TRA | 5.459027 | Aminomethyltransferase folate-binding domain-containing protein [Coccomyxa subellipsoidea C-169] | 1.00E-20 |
|  | CL11131.Contig1_TRA | 4.50676 | hypothetical protein COCSUDRAFT_33358 [Coccomyxa subellipsoidea C-169] | 9.00E-28 |
|  | Unigene1217_TRA | 4.26505 | Cold-shock protein CS120 [Triticum aestivum] | 1.00E-14 |
|  | CL5749.Contig1_TRA | 4.24518 | Retrovirus-related Pol polyprotein from transposon TNT 1-94 [Nicotiana tabacum] | 3.00E-32 |
|  | CL11494.Contig1_TRA | 4.028122 | chlorophyll a/b-binding protein [Coccomyxa subellipsoidea C-169] | 2.00E-66 |
|  | Unigene33926_TRA | 3.945999 | Aminomethyltransferase folate-binding domain-containing protein [Coccomyxa subellipsoidea C-169] | 8.00E-22 |
|  | Unigene15899_TRA | 3.350915 | Leucine-rich repeat extensin-like protein 5 (LRX5) [Arabidopsis thaliana] | 3.00E-08 |
|  | CL7724.Contig2_TRA | 3.033243 | PREDICTED: Vitis vinifera mannan endo-1,4-beta-mannosidase 6-like | 6.00E-20 |
|  | Unigene28552_TRA | 2.950741 | chlorophyll a/b-binding protein [Coccomyxa subellipsoidea C-169] | 1.00E-81 |
|  | Unigene7582_TRA | 2.632276 | hypothetical protein OsJ_25329 [Oryza sativa Japonica Group] | 2.00E-06 |
|  | Unigene24791_TRA | 43.95953 | --- | --- |
|  | Unigene9580_TRA | 26.01251 | --- | --- |
|  | CL13653.Contig1_TRA | 21.81737 | --- | --- |
|  | CL113.Contig2_TRA | 20.18334 | --- | --- |
|  | Unigene24540_TRA | 19.52004 | --- | --- |

Table S7 Genes specifically expressed in CK, LT and TDZ (continued)

|  | **UnigeneID** | **RPKM** | **Annotation** | **E-value** |
| --- | --- | --- | --- | --- |
|  | Unigene9579_TRA | 17.87924 | --- | --- |
|  | CL17628.Contig3_TRA | 15.85356 | --- | --- |
|  | Unigene9793_TRA | 14.59088 | --- | --- |
|  | Unigene34463_TRA | 14.49241 | --- | --- |
|  | Unigene26906_TRA | 14.02637 | --- | --- |
|  | CL5657.Contig4_TRA | 12.69709 | --- | --- |
|  | CL17628.Contig4_TRA | 11.6247 | --- | --- |
|  | Unigene23493_TRA | 11.07869 | --- | --- |
|  | CL18832.Contig2_TRA | 10.00265 | --- | --- |
|  | CL7933.Contig1_TRA | 8.960706 | --- | --- |
|  | Unigene50772_TRA | 8.028792 | --- | --- |
|  | Unigene32335_TRA | 7.834497 | --- | --- |
|  | Unigene15024_TRA | 6.985608 | --- | --- |
|  | Unigene13329_TRA | 6.412773 | --- | --- |
|  | Unigene1464_TRA | 5.365246 | --- | --- |
|  | Unigene49992_TRA | 5.144368 | --- | --- |
|  | Unigene50270_TRA | 4.864894 | --- | --- |
|  | Unigene14302_TRA | 4.421141 | --- | --- |
|  | Unigene13730_TRA | 4.054946 | --- | --- |
| Unigenes specifically expressed in TDZ | Unigene26755_TRA | 32.89011 | Transcription factor RADIALIS (RAD) [Antirrhinum majus] | 2.00E-24 |
|  | Unigene13605_TRA | 5.588997 | starch phosphorylase [Coccomyxa subellipsoidea C-169] | 1.00E-56 |
|  | Unigene16439_TRA | 20.97599 | --- | --- |
|  | Unigene17059_TRA | 14.83302 | --- | --- |
|  | CL5715.Contig1_TRA | 8.726095 | --- | --- |

a. No similarity found.

Table S8 Differential expression of plant hormone associated genes

|  | **UnigeneID** | **Homologs** | **LT** | **TDZ** |
| --- | --- | --- | --- | --- |
| **cytokinin biosynthesis** | Unigene21319_TRA | *AtIPT* | up | down |
|  | Unigene38220_TRA | *AtCYP735A1* | up | -- |
| **cytokinin metabolism** | Unigene45836_TRA | *AtCKX2* | -- | Up |
|  | Unigene36993_TRA | *OsCKX3* | -- | Up |
|  | Unigene36995_TRA | *AtCKX1* | -- | Up |
|  | Unigene36996_TRA | *AtCKX3* | -- | Up |
| **cytokinin signal transduction** | CL17707.Contig1_TRA | *AtARR11* | up | -- |
|  | CL12022.Contig1_TRA | *AtARR9* | -- | up |
|  | CL9210.Contig1_TRA | *AtARR9* | down | -- |
|  | Unigene27329_TRA | *AtARR9* | -- | up |
|  | Unigene43099_TRA | *AtARR6* | down | up |
|  | Unigene43100_TRA | *AtARR9* | -- | up |
|  | Unigene4754_TRA | *AtARR9* | down | up |
| **auxin signal transduction** | Unigene35804_TRA | *OsIAA4* | up | -- |
|  | Unigene40755_TRA | *OsIAA6* | -- | down |
|  | Unigene41593_TRA | *AtARF2* | up | -- |
|  | Unigene43059_TRA | *OsARF22* | up | -- |
|  | CL10310.Contig1_TRA | *AtSKP1A* | -- | up |
|  | CL1488.Contig1_TRA | *AtSKP1B* | -- | down |
|  | Unigene45043_TRA | *AtORE9* | up | -- |
|  | Unigene26995_TRA | *OsGH3.8* | up | -- |
|  | Unigene4369_TRA | *OsGH3.5* | up | up |
|  | CL143.Contig1_TRA | *GmAX6B (SAUR family)* | down | -- |
|  | CL7862.Contig2_TRA | *GmA10A5 SAUR family* | up | -- |
|  | Unigene29691_TRA | *GmAX10A SAUR family* | up | -- |
| **auxin transport** | CL1629.Contig3_TRA | *OsLAX2* | down | -- |
|  | CL492.Contig4_TRA | *OsPIN1C* | up | -- |
| **GA metabolism** | CL2388.Contig3_TRA | *PsGA2ox2* | down | -- |
|  | Unigene31912_TRA | *GA2ox* | down | -- |
|  | Unigene3219_TRA | *AtGA2ox7* | down | down |
| **GA signal transduction** | Unigene31899_TRA | *SlGAI* | up |  |
|  | Unigene29807_TRA | *VvRGL2* | -- | down |
|  | CL10017.Contig1_TRA | *AtPIF4* | -- | up |
|  | Unigene31885_TRA | *AtPIF4* | -- | up |
|  | CL17053.Contig2_TRA | *OsSPL13* | -- | up |
|  | Unigene44647_TRA | *AtSPL5* | -- | up |

Table S9. Arabidopsis mads-box genes used for phylogeny analysis

| **Gene name** | **GI number** |
| --- | --- |
| AtAG | 22328781 |
| AtAGL12 | 862649 |
| AtAGL19 | 30685994 |
| AtAGL6 | 18406848 |
| AtANR1 | 42569026 |
| AtAP1 | 145337310 |
| AtAP3 | 23296739 |
| AtCAL | 145336124 |
| AtFLC | 334187570 |
| AtFLM | 145361961 |
| AtFUL | 145334860 |
| AtMAF2 | 299893471 |
| AtPI | 145358258 |
| AtSEP1 | 186523191 |
| AtSEP2 | 42563430 |
| AtSEP3 | 145336088 |
| AtSEP4 | 145362104 |
| AtSHP1 | 433688783 |
| AtSHP2 | 433688786 |
| AtSOC1 | 145361030 |
| AtSTK | 334186426 |
| AtSVP | 238479325 |

**Fig. S1 The length distribution of assembled *D. nobile* unigenes**


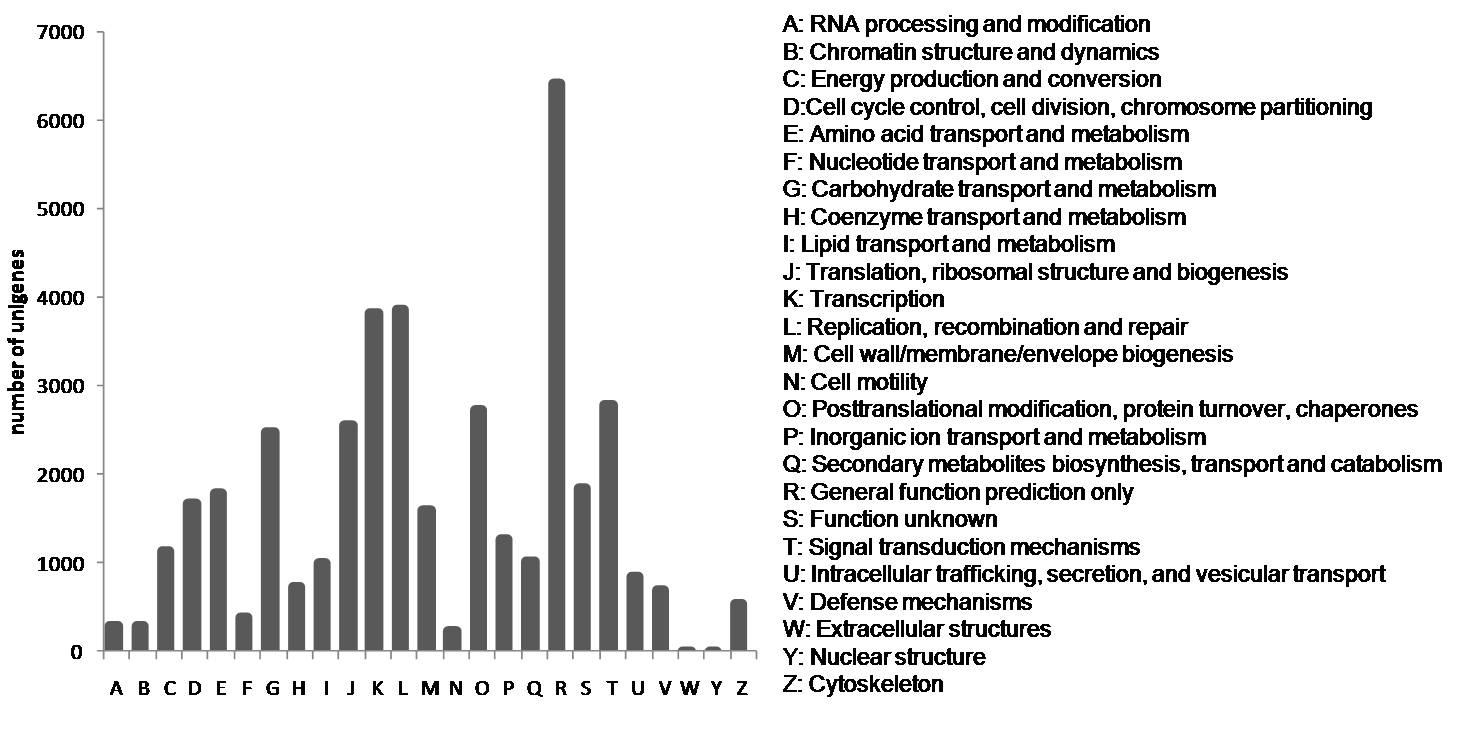


**Fig. S2 COG classification of *D. nobile* unigenes**


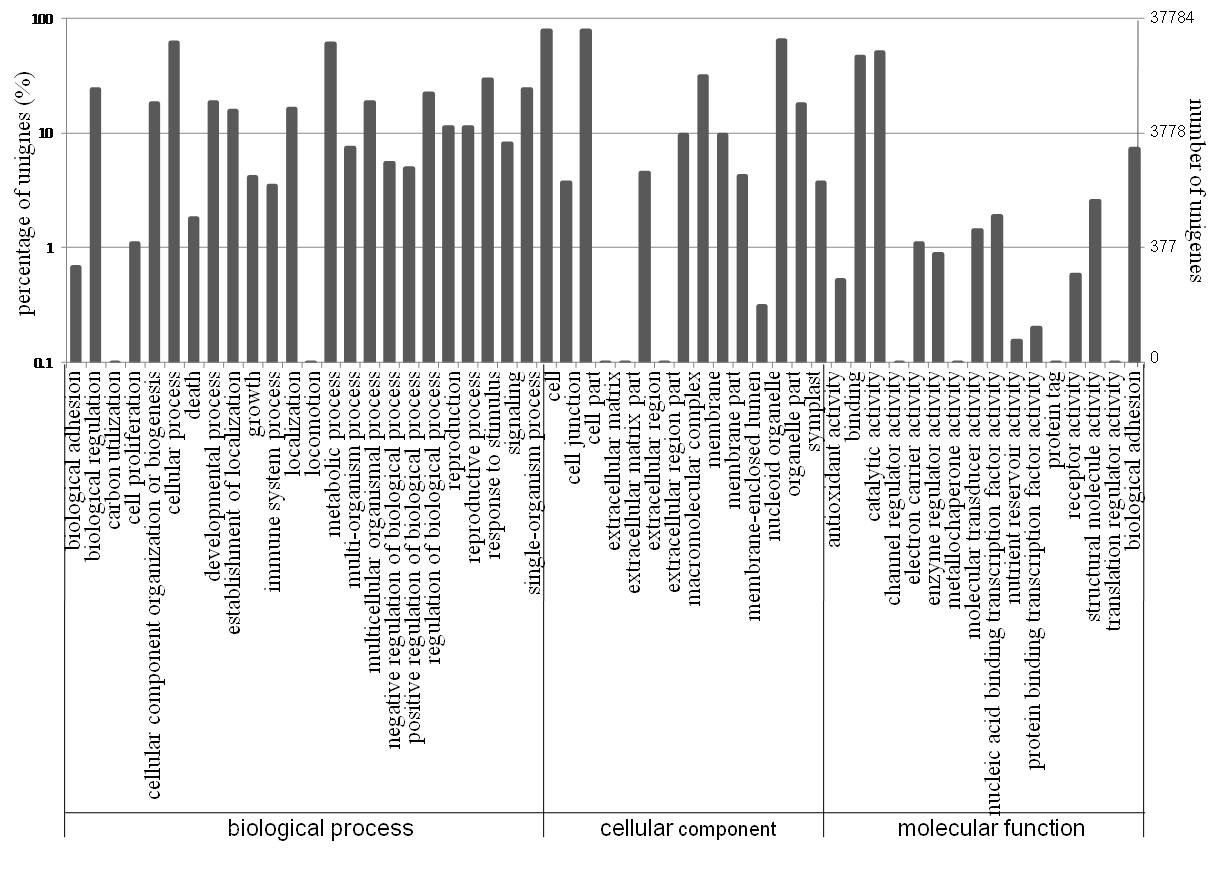
**Fig. S3** **GO classification of *D. nobile* unigenes**


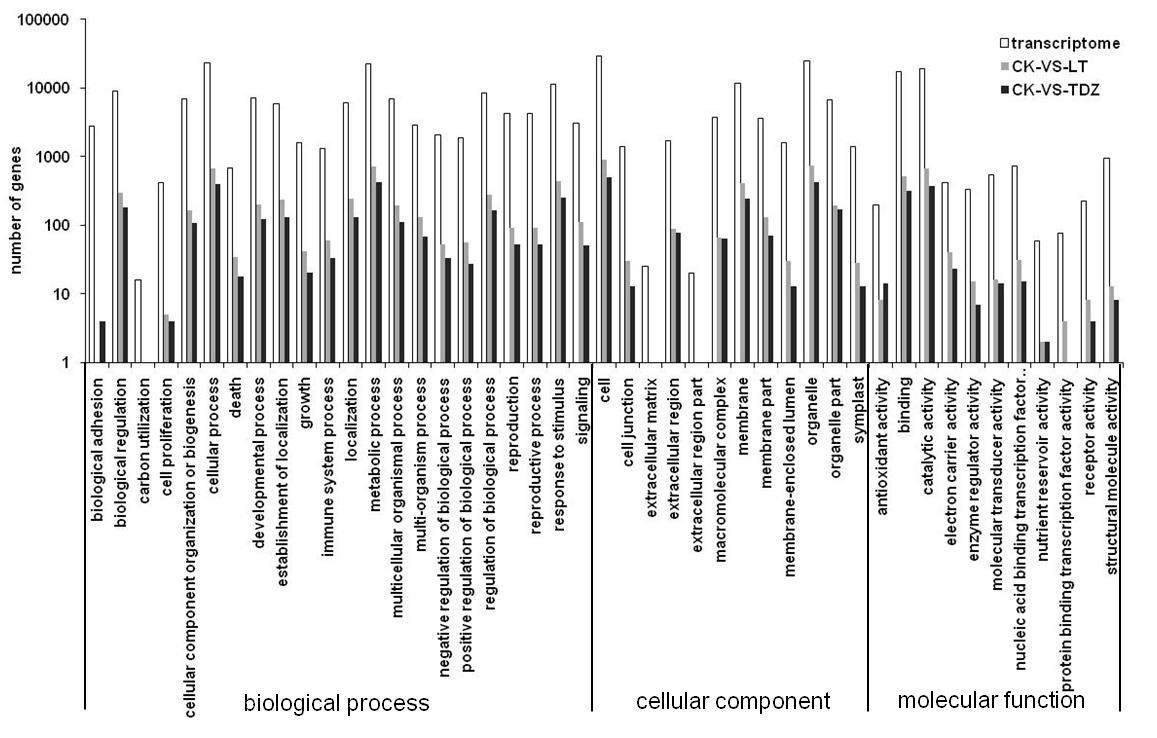


**Fig.S4 GO classification of differentially expressed genes**
